# Head-Down-Tilt Bed Rest With Elevated CO_2_: Effects of a Pilot Spaceflight Analog on Neural Function and Performance During a Cognitive-Motor Dual Task

**DOI:** 10.3389/fphys.2021.654906

**Published:** 2021-08-25

**Authors:** Aditya D. Mahadevan, Kathleen E. Hupfeld, Jessica K. Lee, Yiri E. De Dios, Igor S. Kofman, Nichole E. Beltran, Edwin Mulder, Jacob J. Bloomberg, Ajitkumar P. Mulavara, Rachael D. Seidler

**Affiliations:** ^1^Department of Applied Physiology and Kinesiology, University of Florida, Gainesville, FL, United States; ^2^College of Medicine, University of Florida, Gainesville, FL, United States; ^3^Institute of Aerospace Medicine, German Aerospace Center, Cologne, Germany; ^4^KBR, Houston, TX, United States; ^5^NASA Johnson Space Center, Houston, TX, United States; ^6^Norman Fixel Institute for Neurological Diseases, University of Florida, Gainesville, FL, United States

**Keywords:** spaceflight, dual task, head-down-tilt bed rest (HDBR), CO_2_, spaceflight-associated neuro-ocular syndrome (SANS)

## Abstract

Spaceflight has widespread effects on human performance, including on the ability to dual task. Here, we examine how a spaceflight analog comprising 30 days of head-down-tilt bed rest (HDBR) combined with 0.5% ambient CO_2_ (HDBR + CO_2_) influences performance and functional activity of the brain during single and dual tasking of a cognitive and a motor task. The addition of CO_2_ to HDBR is thought to better mimic the conditions aboard the International Space Station. Participants completed three tasks: (1) COUNT: counting the number of times an oddball stimulus was presented among distractors; (2) TAP: tapping one of two buttons in response to a visual cue; and (3) DUAL: performing both tasks concurrently. Eleven participants (six males) underwent functional MRI (fMRI) while performing these tasks at six time points: twice before HDBR + CO_2_, twice during HDBR + CO_2_, and twice after HDBR + CO_2_. Behavioral measures included reaction time, standard error of reaction time, and tapping accuracy during the TAP and DUAL tasks, and the dual task cost (DTCost) of each of these measures. We also quantified DTCost of fMRI brain activation. In our previous HDBR study of 13 participants (with atmospheric CO_2_), subjects experienced TAP accuracy improvements during bed rest, whereas TAP accuracy declined while in the current study of HDBR + CO_2_. In the HDBR + CO_2_ subjects, we identified a region in the superior frontal gyrus that showed decreased DTCost of brain activation while in HDBR + CO_2_, and recovered back to baseline levels before the completion of bed rest. Compared to HDBR alone, we found different patterns of brain activation change with HDBR + CO_2_. HDBR + CO_2_ subjects had increased DTCost in the middle temporal gyrus whereas HDBR subjects had decreased DTCost in the same area. Five of the HDBR + CO_2_ subjects developed signs of spaceflight-associated neuro-ocular syndrome (SANS). These subjects exhibited lower baseline dual task activation and higher slopes of change during HDBR + CO_2_ than subjects with no signs of SANS. Collectively, this pilot study provides insight into the additional and/or interactive effects of CO_2_ levels during HDBR, and information regarding the impacts of this spaceflight analog environment on the neural correlates of dual tasking.

## Introduction

Prolonged exposure to microgravity has widespread impacts on human physiology and performance. As space flights become longer and more frequent, it is becoming increasingly important to characterize the pattern and persistence of microgravity-induced changes in cognitive and sensorimotor processing. Cognitive-motor dual tasking, which involves the concurrent performance of both a cognitive and a motor task, is common in daily life and is of high operational relevance for astronauts. For example, during extravehicular activities, an astronaut is required to move while also attending to verbal directions. Declines in performance are often evident when people perform under dual as opposed to single task conditions (Schaefer and Schumacher, 2011). Dual task cost (DTCost), a measure of this dual task performance decrement, is a sensitive indicator of generalized cognitive-motor capacity (Tombu and Jolicoeur, 2003; Verhaeghen et al., 2003; Riby et al., 2004; Hartley et al., 2011) that has been used to monitor spaceflight-related changes in cognition (Manzey, 2000) and brain activation (Yuan et al., 2016). Dual tasking typically requires activation of all the brain regions involved in component cognitive and motor tasks, along with additional prefrontal areas that are not engaged during these single tasks (D’Esposito et al., 1995; Adcock et al., 2000; Szameitat et al., 2002). Some evidence also exists for increased amplitude of activation in frontal (Herath et al., 2001; Szameitat et al., 2002; Erickson et al., 2007; Dux et al., 2009; Holtzer et al., 2011) and occipital (Hartley et al., 2011) regions during dual task relative to single motor tasks. These activation increases may be proportional to the complexity of the two tasks being performed (Mirelman et al., 2014).

Environmental stressors, including many specific conditions of spaceflight, decrease overall attentional capacity (Hockey, 1986) and may affect the ability to attend to interfering stimuli. During spaceflight, dual task performance has been shown to decline more than single task performance, increasing DTCost (Manzey et al., 1995; Manzey and Lorenz, 1998b; Bock et al., 2010). These decrements are primarily in motor tracking performance (in both single and dual tasks), whereas cognitive task performance during spaceflight shows little decline during single or dual tasks (Manzey et al., 1995, 1998; Lathan and Newman, 1997; Strangman et al., 2014; Tays et al., 2021). Short and long-duration spaceflight investigations have observed initial declines in single and dual task visuomotor performance, followed by recovery of performance while still in spaceflight (Manzey et al., 1995, 1998), indicating a potential adaptation of performance to the spaceflight environment. During a short duration flight (8 days in space), one astronaut’s performance of both single and dual tasks of varying difficulty declined, then returned to baseline levels after 2 days of flight. Performance worsened again on day four of flight, before gradually returning to baseline following their return to Earth (Manzey et al., 1995). A case study of a different single astronaut during a long-duration spaceflight (438 days in space) showed a similar initial pattern of decline in single and dual task performance. In this case, performance returned to baseline after 3 weeks and remained at this level for the remainder of the mission. After returning to Earth, the subject experienced another adjustment period, characterized by initially worsening motor performance (in both single and dual tasks) with subsequent recovery toward pre-flight levels during the following 2 weeks (Manzey et al., 1998). This raises questions not only of recovery after returning to Earth, but also of adaptation during spaceflight. Our understanding of the effects of spaceflight on cognitive-motor dual tasking remains largely based on these performance-based case studies, in part due to the difficulty of performing assessments in space. In this investigation, we hypothesized that in-bed rest recovery effects – as seen in these behavioral studies during spaceflight– would also be shown in fMRI metrics of neural activation.

Head-down-tilt bed rest (HDBR) is commonly used to simulate several effects of spaceflight, including axial body unloading and the headward shift of cerebrospinal fluid within the skull (At’kov and Bednenko, 1992; Caprihan et al., 1999; Koppelmans et al., 2016, 2017), while also allowing for multimodal assessments that cannot be performed in space. The effects of HDBR on the performance of cognitive-motor dual tasking performance and on brain activation are not well understood. Our previous longitudinal assessment of dual tasking during 70 days of HDBR identified both increases and decreases in brain activation with dual tasking (Yuan et al., 2016). Several of these changes were positively correlated with change in reaction time during single task. Additionally, we found negative correlations between single task reaction time and brain activation in the cerebellum and brainstem. Furthermore, we found a positive correlation between DTCost of brain activation and DTCost of reaction time throughout the brain. Subjects who had the greatest increases in DTCost of reaction time also had the greatest increases in DTCost of brain activation (Yuan et al., 2016). These relationships between single and dual task performance and brain activation suggest that HDBR induces an incomplete compensatory neural response.

In addition to microgravity, other aspects of the International Space Station (ISS) environment may affect human physiology, including the elevated carbon dioxide (CO_2_) levels. In the enclosed and isolated environment of the ISS, the partial pressure of CO_2_ peaks at 8.32 mmHg (Law et al., 2014), and the average pCO_2_ is close to 3.5 mmHg (0.46% CO_2_), which is about 10 times higher than ambient levels on Earth. CO_2_ affects cerebral blood flow and blood pH (Brian, 1998), as well as ventilation rate (Castro and Keenaghan, 2019), and generalized physiological stress in the form of increased polymorphonuclear cell levels (Marshall-Goebel et al., 2017). These changes in cerebral blood flow, oxidative stress, and oxygen levels (Battisti-Charbonney et al., 2011) could have significant effects on the metabolic productivity of neural tissue, thereby altering its functional output. Past work has demonstrated that motor performance is altered with 26 days of exposure to elevated CO_2_ (Manzey and Lorenz, 1998a), of either 0.7 or 1.2%. Exposure to increased levels of CO_2_ also inhibits blood oxygen level dependent (BOLD) signaling mechanisms (Xu et al., 2011) that are measured by functional magnetic resonance imaging (fMRI), suggesting that CO_2_ levels could directly impact the findings of fMRI studies of dual tasking. A previous HDBR + CO_2_ study, the SPACECOT pilot campaign, involved short duration (26.5 h) exposure to 0.5% CO_2_ during 12° HDBR (Marshall-Goebel et al., 2017). The investigators reported that elevation of ambient CO_2_ may actually mitigate some of the motor decrement associated with HDBR (Basner et al., 2018). The SPACECOT project was, however, limited by the short intervention duration and its small sample size (*n* = 6) of exclusively male subjects, one of whom also had previous HDBR experience. Nonetheless, these findings suggest that HDBR in elevated CO_2_ affects motor performance. Thus, the goals of the present work included characterization of the performance and neural correlates of a cognitive-motor dual task during HDBR + CO_2_, as well as the time courses that they follow. We also compared the results of the HDBR + CO_2_ study to findings from our previous study of HDBR in atmospheric CO_2_ levels. This comparison is exploratory, however, given several differences between the two studies (e.g., exposure duration, sex distribution, participants’ exercise levels, location of intervention etc.). In light of prior findings, we hypothesized that the addition of CO_2_ to a standard HDBR intervention would mitigate behavioral changes seen with dual tasking, as well as alter the functional neural response to HDBR.

Over 60% of ISS astronauts return to Earth presenting with at least one sign of spaceflight-associated neuro-ocular syndrome (SANS), a collection of structural and functional ophthalmological changes considered to be one of the greatest challenges for deep space travel to overcome (Brunstetter, 2017). It has been suggested that the development of SANS could be linked to elevated CO_2_ levels (Law et al., 2014). Previous bed rest interventions have not replicated the signs of SANS or the changes in intracranial and intraocular pressure that are seen in astronauts when they return from spaceflight (Brunstetter, 2017; Basner et al., 2018). This could be due, in part, to the short duration of past interventions because signs of SANS are more prevalent after missions of 5–6 months than after shorter shuttle missions (Alperin and Bagci, 2018). In the present work, 5 of the 11 bed rest subjects developed signs of SANS (Laurie et al., 2019). Although the interactions of SANS with cognitive and sensorimotor behavior are not well-investigated, we recently reported that these five individuals with signs of SANS had slower reaction time than the six individuals who did not present with signs of SANS (Lee et al., 2019a). Thus, in the present work, we also include exploratory analyses to characterize potential effects of SANS status on brain activation during dual tasking across the HDBR + CO_2_ intervention.

## Materials and Methods

### Participants

#### HDBR + CO_2_ Participants

Eleven healthy subjects (six males) participated in the HDBR + CO_2_ campaign. Subjects were aged 33.9 ± 8.03 years at the time of admission (Table 1). Subjects had a mean height of 173.8 (ranging from 158 to 186) cm and mean weight of 70.8 (ranging from 55 to 84) kg. The study took place at the :envihab facility at the German Aerospace Center (*Deutsches Zentrum für Luft- und Raumfahrt*, DLR) in Cologne, Germany as a part of the larger Visual Impairment Intracranial Pressure and Psychological :envihab Research (VaPER) campaign. Participants were required to pass an Air Force Class III equivalent physical examination prior to enrollment. Participants were excluded if they had any pre-existing conditions or medication use. After HDBR + CO_2_, five of the 11 subjects presented signs associated with SANS (Laurie et al., 2019). As a part of the NASA standard measures assessments, participants also had blood drawn 3 days before HDBR + CO_2_ and on the day of reambulation following HDBR + CO_2_. Standard arterial blood gas measurements were collected to determine the arterial partial pressure of carbon dioxide (P_a_CO_2_) at each of these times. Sleep data from the HDBR + CO_2_ cohort in comparison to a 60-day bed rest control has also been reported (Basner et al., 2021), with no changes in sleep duration between the two groups. Average sleep duration was 7.54 h (7.31–7.76 95% CI) in the control group and 7.51 h (7.32–7.70 95% CI) in the HDBR + CO_2_ group. During their stay, subjects were assessed clinically and given any indicated medications. It was determined that none of these medications affected task performance. A supplementary medication review can be accessed, along with all other supplementary material at https://www.frontiersin.org/articles/10.3389/fphys.2021.654906/full#supplementary-material.

**TABLE 1:** Subject demographic information.

	**70-day HDBR**	**30-day HDBR + CO_2_**	
Number of Participants	*n* = 13 (13 Males, 0 Females)	*n* = 11 (6 Males, 5 Females)**	
Mean Age* (Standard Deviation)	29.34 (3.23)	33.91 (8.03)	
		SANS	Non-SANS
Number of Participants		*n* = 5 (2 Males, 3 Females)	*n* = 6 (4 Males, 2 Females)
Mean Age* (Standard Deviation)		37.77 (7.5)	30.68 (7.5)

Demographic information is presented only for subjects who were not excluded from analyses due to outliers or head movement. We separate demographic information by intervention (i.e., HDBR + CO_2_ or HDBR) and by SANS status (i.e., those who either did or did not present signs of spaceflight-associated neuro-ocular syndrome (SANS) after HDBR+CO_2_). *Age in Years. **n = 10 for Brain/Behavior Correlations (5 Males, 5 Females).

#### HDBR Participants

Eighteen healthy male subjects participated in our previous 70-day HDBR campaign at the University of Texas Medical Branch, Galveston, TX, United States. Five subjects were removed from analyses due to excessive head motion or beta contrast value outliers, leaving thirteen subjects included in HDBR analyses. All participants were right-handed and aged 29.3 ± 3.2 years at the time of admission (Table 1) and passed an Air Force Class III equivalent physical examination before admission. Subjects had a mean height of 177.2 (ranging from 166.5 to 187) cm and mean weight of 78.2 (ranging from 58 to 97.2) kg. All 13 subjects engaged in supine exercise during HDBR as part of a concurrent study. These subjects, herein referred to as “HDBR” subjects to differentiate them from HDBR + CO_2_ subjects, are included as a comparator group to test for differential or additive effects of the HDBR + CO_2_ intervention. We have previously published dual task brain and behavioral results from the HDBR cohort alone (Yuan et al., 2016).

#### Ethics Approval

Both studies were conducted in accordance with the Declaration of Helsinki and were approved by the Ethical Commission of the Ärztekammer Nordrhein (HDBR + CO_2_) and the Institutional Review Boards of the University of Florida (HDBR + CO_2_), University of Michigan (HDBR), University of Texas-Medical Branch (HDBR), and NASA Johnson Space Center (HDBR and HDBR + CO_2_). Written informed consent was obtained from all participants. Participants received monetary compensation.

### Bed Rest Protocols

#### HDBR + CO_2_ Protocol

HDBR + CO_2_ participants experienced 6° HDBR combined with an ambient CO_2_ partial pressure of 3.8 mmHg (∼0.5%) for 30 days. This increased level of CO_2_ was intended to simulate the elevated CO_2_ concentration onboard the ISS (Law et al., 2014). Although 0.5% CO_2_ does not typically cause symptoms on Earth, astronauts onboard the ISS often report headaches and visual disturbances (Law et al., 2014), as well as impairments in cognitive performance (Satish et al., 2012) that correlate with CO_2_ levels. This may result from the combined effects of elevated CO_2_ with headward fluid shifts, which also occur in bed rest. CO_2_ was administered via room-wide elevations in concentration. Rooms were controlled at a temperature of 22°C and 30–45% humidity. The slight decrease in atmospheric pO_2_ that accompanied an increase in pCO_2_ had no effect on the participants’ arterial pO_2_. During arterial blood draws, subjects were instructed not to speak and to breathe normally. Blood draws were conducted in the seated position prior to or following the intervention. During bed rest, they were conducted in the 6° head-down-tilt position. Divergent blood gas measurements were repeated as needed.

HDBR + CO_2_ subjects were required to keep at least one shoulder in contact with the mattress at all times and were permitted to use a pillow only while on their side. Subjects were instructed not to stretch, contract, or raise their legs during HDBR + CO_2_. Physical therapy sessions occurred every other day during the intervention to avoid muscle tightness and provide comfort to the subjects. Participants were highly compliant as a result of 24/7 staff and video monitoring.

**FIGURE 1:**
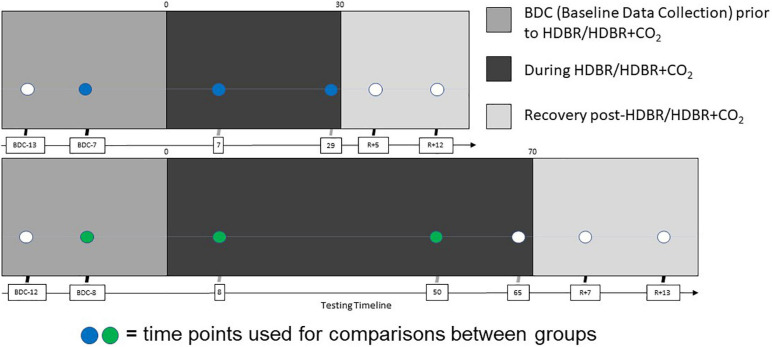
Testing timeline. (Top) Testing timeline for HDBR + CO_2_ intervention. Two initial acquisitions sessions took place 13 and 7 days prior to bedrest (BDC-13 and BDC-7), two more were made on the 7th and 29th days of the HDBR + CO_2_ intervention, and the final two were made 5 and 12 days after the end of bedrest (R+5 and R+12). (Bottom) Testing timeline for HDBR intervention. HDBR acquisition occurred at seven sessions: 14.1 ± 3.8 days and 7.9 ± 2.0 days before the start of HDBR, 8.4 ± 1.0 days, 50.6 ± 0.9 days, and 66.8 ± 1.8 days after the onset of HDBR, as well as 6.7 ± 0.8 days and 11.4 ± 1.6 days after HDBR (Yuan et al., 2016). Each time point for both interventions included cognitive-motor dual task behavioral testing with concurrent fMRI acquisition. The sessions used for intercept, slope, and normalized slope comparisons between groups are indicated in blue (HDBR + CO_2_) and green (HDBR).

We collected fMRI and behavioral data at six sessions: twice before the start of HDBR + CO_2_, twice during HDBR + CO_2_, and twice after HDBR + CO_2_ (Figure 1). Subjects maintained the head-down-tilt position in the MRI scanner by laying on a foam wedge; the head was supine within the MRI head coil.

#### HDBR Protocol

Head-down-tilt bed rest participants were exposed to 6° of HDBR for 70 days. Throughout the intervention, subjects remained in bed rest for 24 h a day. Subjects were also not permitted to raise their head or prop themselves up, except for 30 min at each meal when they were allowed to support their head with their hand. While in HDBR + CO_2_, subjects were not permitted to support their head with their hand at any time (including during meals).

We collected fMRI data at seven time points: twice before the start of HDBR, three times during HDBR, and twice after HDBR (Yuan et al., 2016; Figure 1). Subjects maintained the 6° head-down-tilt position in the MRI scanner by laying on a foam wedge; the head was supine within the MRI head coil.

### MRI Image Acquisition

#### HDBR + CO_2_

At each time point, HDBR + CO_2_ subjects underwent an identical scan protocol using the same 3-Tesla Siemens BioGraph mMR MR-PET scanner. For each fMRI run, we used a gradient echo T2^∗^-weighted echo-planar imaging (EPI) sequence with the following parameters: TR = 2500 ms, TE = 32 ms, flip angle = 90°, FOV = 192 mm × 192 mm, slice thickness = 3.5 mm, matrix = 64 × 64, voxel size = 3.0 mm × 3.0 mm × 3.5 mm, 37 axial slices. Each 260-s run was acquired in 104 consecutive functional volumes. We also collected a T1-weighted gradient-echo pulse sequence during each acquisition using the following parameters: TR = 1900 ms, TE = 2.44 ms, flip angle = 9°, FOV = 250 mm × 250 mm, slice thickness = 1.0 mm; matrix = 512 × 512, voxel size = 0.49 mm × 0.49 mm × 1 mm, 192 sagittal slices; duration = ∼4 min. All acquired data is available in a central NASA repository and is available upon request to the NASA data archives.

#### Head-Down-Tilt Bed Rest

At each time point, HDBR subjects underwent an identical scan protocol using the same 3-Tesla Siemens Magnetom Skyra MRI scanner. For each fMRI run, we used a gradient echo T2^∗^-weighted EPI sequence with the following parameters: TR = 3660 ms, TE = 39 ms, flip angle = 90°, FOV = 240 mm × 240 mm, slice thickness = 4 mm, slice gap = 1 mm, matrix = 94 × 94, voxel size = 2.55 mm × 2.55 mm × 5.0 mm, 36 axial slices. Each 260-s run was acquired in 71 consecutive functional volumes. We also collected a T1-weighted gradient-echo pulse sequence at each time point using the following parameters: TR = 1900 ms, TE = 2.49 ms, flip angle = 9°, FOV = 270 mm × 270 mm, slice thickness = 0.9 mm; matrix = 288 × 288, voxel size = 0.94 mm × 0.94 mm × 0.9 mm, 192 sagittal slices; duration = ∼4 min. All acquired data is available in a central NASA repository and is available upon request to the NASA data archives.

### Dual Task Assessment

**FIGURE 2:**
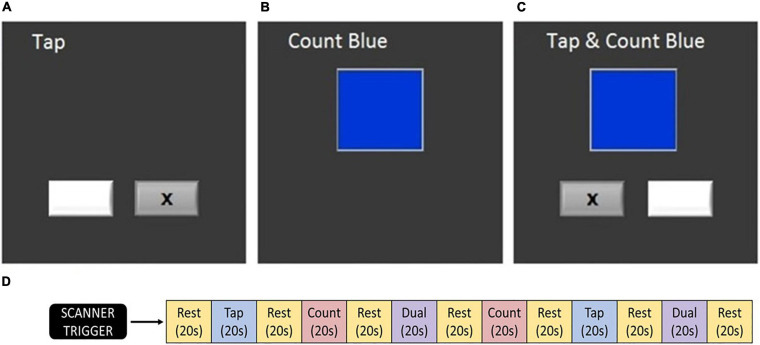
Testing paradigm. **(A)** Tapping task, **(B)** counting task, **(C)** dual task, and **(D)** order of task blocks (Yuan et al., 2016). At each testing session, this sequence was conducted twice. Participants completed these tasks during fMRI acquisition.

During each fMRI acquisition, participants completed the dual task assessment (Figure 2). This task was identical across time points and between the HDBR + CO_2_ and HDBR cohorts. Participants completed three tasks: (1) TAP, (2) COUNT, and (3) DUAL. During the TAP task, two boxes were presented on the screen. Every 800 ms, an “x” was presented in one of the two boxes, indicating which of two adjacent buttons the participants were to press with their left or right index finger. During the COUNT task, a stimulus box was presented that changed color at a rate of 3 Hz. Subjects counted the number of times the box turned blue. The box only turned blue approximately four times out of 60 stimuli to ensure that subjects were remaining attentive. During the DUAL task, subjects performed the TAP and COUNT tasks simultaneously. The COUNT stimulus box was centered above the two TAP stimuli boxes to minimize eye movements.

During each fMRI run, blocks of each task (TAP, COUNT, and DUAL) were presented in a pseudo-randomized order. Twenty second rest periods were included before the first block, after the final block, and between each block, constituting an fMRI run of 260 s. Participants completed two fMRI runs on each test date. Recorded measures included: the reaction time from TAP stimulus onset to motor response and the percent of targets correctly responded to in both the TAP and COUNT tasks. Verbal responses for each of the two COUNT and DUAL blocks were reported following each run. Measures were collected for the TAP, COUNT, and DUAL tasks and used to calculate the DTCost of each measure. DTCost was calculated as the percent change in performance measures from single to dual task. Each measure and their DTCost were analyzed as dependent measures of task performance.

### Statistical Analyses of P_a_CO_2_ and Behavioral Data

#### HDBR + CO_2_ Effects on P_a_CO_2_

We conducted all analyses of behavioral performance using R version 3.6.0 (R Core Team, 2013). First, we conducted a paired *t*-test (one-tailed) to test for changes in P_a_CO_2_ from pre- to post-HDBR + CO_2_. We defined statistical significance as *p* < 0.05.

#### Effect of HDBR + CO_2_ and HDBR on Performance

We used linear mixed models to assess the effects of HDBR + CO_2_ and HDBR on task performance. For these models, we analyzed accuracy, reaction time, and standard error of reaction time during the TAP and DUAL tasks, along with the DTCost of all three measures. We tested for a significant effect of time during bed rest in both the HDBR + CO_2_ and HDBR cohorts, as well as for significant differences in this effect between cohorts. We excluded behavioral measurements from the first session for all subjects in both cohorts to control for learning effects between the first two sessions, as has been done in our previous analyses (Yuan et al., 2016). Thus, we used data from sessions 2–4 for HDBR+CO_2_ and sessions 2–5 for HDBR to test for a main effect of each bed rest intervention. In each case, we entered time as the number of days elapsed since session 2 and treated time as a continuous variable.

We controlled for the effects of age and sex in all models including the HDBR + CO_2_ cohort. We controlled for the effects of age but not sex in models that only involved the HDBR cohort because all of these subjects were male. In the analyses between the HDBR and HDBR + CO_2_ cohorts, we also controlled for the effects of the HDBR exercise intervention. For each model described here, we used restricted maximum likelihood (REML) estimation, with statistical significance defined as *p* < 0.05.

#### Recovery of Performance

We conducted linear mixed models to test for recovery after each bed rest intervention. For these recovery models, we used data from sessions 4–6 for HDBR+CO_2_ and from sessions 5–7 for HDBR. We tested for significant recovery effects in the HDBR and HDBR + CO_2_ cohorts, as well as the SANS and non-SANS HDBR + CO_2_ cohorts. We also tested for significant differences in these recovery patterns between the HDBR + CO_2_ and HDBR cohorts, and between the SANS and non-SANS HDBR + CO_2_ cohorts.

Of note, we previously reported behavioral analyses for both the HDBR + CO_2_ (Lee et al., 2019a) and HDBR cohorts (Yuan et al., 2016). However, these analyses did not examine recovery patterns or the DTCost of behavioral measures. The previous analyses also included the two HDBR subjects who were removed from the present analyses due to head movement during the dual task fMRI scans. For each model described here, we used restricted maximum likelihood (REML) estimation, with statistical significance defined as *p* < 0.05.

#### Effect of SANS Status on Performance

We ran linear mixed models to test for significant changes in performance in subjects who presented with signs of SANS and those that did not present with signs of SANS after HDBR + CO_2_. We also ran linear mixed models to compare these performance changes between these cohorts. Accuracy, reaction time, and standard error of reaction time, as well as the DTCost of all three measures were assessed in these analyses. For each model described here, we used restricted maximum likelihood (REML) estimation, with statistical significance defined as *p* < 0.05.

### Functional Image Preprocessing and Statistical Analyses

#### Whole Brain Preprocessing

We processed the dual task data from the HDBR and HDBR + CO_2_ subjects using identical procedures to our past work (Hupfeld et al., 2020a; Salazar et al., 2020, 2021) using the established Statistical Parametric Mapping software (SPM12, version 7219) (Ashburner et al., 2016) and pipeline (Penny et al., 2011; Ashburner et al., 2016) through MATLAB R2016a (version 9.0). We first corrected functional images for slice timing and realigned the images to correct for volume-to-volume head motion. We then normalized the images to the Montreal Neurological Institute (MNI) 152 space template (Friston et al., 1995) using the following procedure: (1) we reset the origin of all T1-weighted structural images to the anterior commissure; (2) we coregistered the T1-weighted structural images to the corresponding mean functional image; (3) we segmented the T1-weighted images by tissue type (i.e., gray matter, white matter, cerebrospinal fluid, bone, soft tissue, and air) using the SPM12 Dartel algorithm with a sampling distance of 3 mm; (4) we applied the resulting forward deformation fields to the T1 and functional images to warp each into MNI 152 space using 7th degree B-spline normalization.

After normalization, we used the Artifact Detection Tool (ART) to detect outliers of global brain intensity (Z threshold >9) or head movement greater than 3 mm. We removed volumes exceeding these thresholds from the functional timeseries images for each cohort. For the HDBR + CO_2_ cohort we removed volumes from three subjects in total due to global intensity outliers: volumes 1–2 of session 1-run 2 (i.e., REST volumes) for two subjects; volumes 52–71 (i.e., the last 18 volumes) of session 3-run 2 (i.e., 10 REST volumes; 8 DUAL volumes) for one subject. HDBR + CO_2_ subjects moved very little, so no volumes were excluded due to movement. For the HDBR cohort, we removed two subjects from subsequent analyses entirely due to global intensity and movement outliers. We also removed volumes from two additional HDBR subjects due to head movement: volumes 1–19 of session 2-run 2 for one subject (i.e., 11 REST volumes, 6 TAP volumes, and 2 COUNT volumes); session 2-run 1 all volumes and session 2-run 2 volumes 1-31 (i.e., 17 REST volumes, 5 TAP volumes, 5 COUNT volumes, and 4 DUAL volumes). After these exclusions, we spatially smoothed the functional images using an 8-mm, full-width, half-maximum, three-dimensional Gaussian kernel.

#### Whole Brain Subject-Level Statistical Analyses

At the single subject level, we calculated voxelwise brain activity for the TAP, COUNT, and DUAL tasks compared to rest. We then calculated images for the DTCost of brain activation by subtracting the average of the activation during the TAP and COUNT single tasks from activation during the DUAL task. We used a masking threshold of “-Inf” and applied the SPM intracranial volume mask (mask_ICV.nii) to circumvent SPM’s default intensity-based threshold for general linear model inclusion (≥80% of average global intensity). In each subject-level model, we included the ART volume-to-volume head motion parameters as nuisance regressors.

#### Cerebellar Preprocessing and Subject-Level Statistical Analyses

Montreal Neurological Institute template-based normalization does not accurately warp the cerebellum (Diedrichsen, 2006; Diedrichsen et al., 2009). Instead, identical to our past work (Hupfeld et al., 2020a; Salazar et al., 2020, 2021), we implemented a combination of two optimized cerebellar processing pipelines, CEREbellum Segmentation (CERES) (Romero et al., 2017) and the Spatially Unbiased Infratentorial Template (SUIT) (Diedrichsen, 2006; Diedrichsen et al., 2009). We used CERES to extract and segment the cerebellum, according to a cerebellum-specific tissue type probability map and the T1-weighted image from each subject and session. We elected to use CERES for cerebellar extraction because this pipeline performed more accurately than SUIT. CERES processing resulted in gray and white matter masks, as well as an overall cerebellar mask, specific to each T1-weighted acquisition. We then used the SUIT Dartel normalization algorithm (suit_normalize_dartel) to obtain the affine transformation matrix and flowfield to warp each subject/session structural image from native space to SUIT template space.

We conducted whole-brain slice timing and volume-to-volume realignment as previously described. We then coregistered these functional images to the T1-weighted image that was segmented using CERES and conducted subject-level statistical analyses, also as previously described. We applied the affine transformation matrices, flowfields, and subject/session-specific cerebellar masks to the subject-level contrast images to reslice these images into SUIT template space using the (suit_reslice_dartel) function. We applied a 2-mm smoothing kernel because of the comparatively small volume of the cerebellum. We then conducted group-level statistical analyses on these subject-level statistical images, as well as on the previously described whole brain contrast images. For cerebellar group-level analyses, we applied a binary mask of the SUIT.nii template to exclude any activation that spilled off of the cerebellum due to smoothing.

### Group-Level fMRI Statistical Analyses

All neuroimaging statistical analyses were thresholded at *k* = 10 voxels for the whole brain analyses and at *k* = 6 for the cerebellar analyses. We defined statistical significance as *p* < 0.0001 for all models, except the proof-of-concept main effect model (see section “Neural Correlates of Dual Task Performance at Baseline”) and the exploratory SANS versus non-SANS model (see section “SANS vs. non-SANS Comparisons Within the HDBR + CO_2_ Cohort”), for which statistical significance was defined as *p* < 0.001. We first applied family-wise error rate (FWE) < 0.05 (Ostwald et al., 2018) and false discovery rate (FDR) < 0.05 (Chumbley et al., 2010) statistical corrections. However, these corrections yielded no significant clusters of activation for any of the analyses below, likely due to the small sample size of the study.

We conducted each of these group-level analyses for TAP, COUNT, DUAL, and DTCost. Here, we report only DTCost fMRI results, except for the proof-of-concept main effect (see section “Neural Correlates of Dual Task Performance at Baseline”) and exploratory SANS analyses (see section “SANS vs. non-SANS Comparisons Within the HDBR + CO_2_ Cohort”), in which we reported results for all tasks and DTCost. For each model, we included age and sex as covariates of no interest.

#### Neural Correlates of Dual Task Performance at Baseline

To assess the main effect of TAP, COUNT, DUAL, and DTCost versus values at rest, we calculated average brain activation for each task using a one-sample *t*-test of the scans from session 2. The purpose of this test was only to confirm that we obtained activation in the expected brain regions during each task.

#### Time Course of Change in Neural Response to HDBR + CO_2_

**FIGURE 3:**
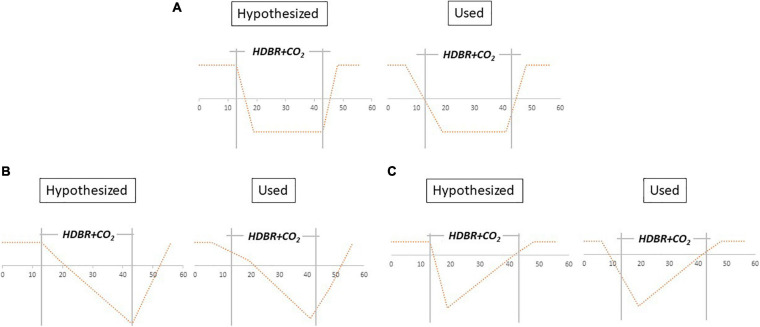
Hypothesized models vs. tested contrast vectors. **(A)** Instant decrease, **(B)** cumulative decrease, and **(C)** instant decrease with recovery in- HDBR + CO_2_. Models were also applied for instant increase, cumulative increase, and instant increase with recovery in- HDBR + CO_2_.

We conducted linear mixed model analyses using the flexible factorial model within SPM to assess longitudinal patterns of change in brain activation during the HDBR + CO_2_ study. We created contrast vectors to test several hypothesized models of change from session 2 to session 6. The applied contrast vectors reflected three hypothetical patterns of change: “instant change,” “cumulative change,” and “instant change with recovery in HDBR + CO_2_” (Figure 3). We tested both positive and negative change during HDBR+CO_2_ for each hypothesized model. Instant and cumulative change models of positive and negative change have been previously applied to HDBR data, as reported by Yuan et al. (2016). (1) The instant change model reflects a response that is dependent only on bed rest status. Once bed rest begins, there is a change from baseline that remains constant and then recovers when the intervention concludes. (2) The cumulative change model reflects a time-dependent pattern in which brain changes become greater over the course of bed rest. (3) The instant change with recovery in HDBR + CO_2_ model reflects the same response as the initial change model, but instead of maintaining the change throughout the intervention, the change begins to return toward baseline levels while still in bed rest. This is reflective of a gradual adaptation to bed rest. These three hypothesized models involve change at the start of the bed rest intervention; however, because MRI acquisitions did not occur on the first or last day of HDBR + CO_2_, the applied models are slightly imperfect representations of the hypothesized patterns of change (Figure 3). Here we defined statistical significance as *p* < 0.0001.

#### Correlations Between Behavioral and Neural Changes Associated With HDBR + CO_2_

To obtain measures of the magnitude of change that occurred during the HDBR + CO_2_ intervention, we subtracted each subject’s fMRI statistical contrast image from session 2 from their contrast image from session 4. We also calculated behavior change scores by subtracting session 2 scores from session 4 scores. We then tested correlations between pre- to post-HDBR + CO_2_ brain activation change and pre- to post-HDBR + CO_2_ behavioral change. For these models, we used non-parametric one-sample *t*-tests with one covariate of interest via the SnPM toolbox (Nichols and Holmes, 2002). We performed 15,000 permutations and set variance smoothing = 8 mm for the whole brain analyses and variance smoothing = 2 mm for the cerebellar analyses. We excluded one of the 11 HDBR + CO_2_ subjects from these brain/behavior correlations because of missing behavioral data from session 2.

#### Comparisons of Changes Associated With HDBR and HDBR + CO_2_

To characterize the differential effects of HDBR with and without elevated CO_2_, we calculated baseline (i.e., intercept) and slope of change statistical images for each subject of both the HDBR + CO_2_ and HDBR studies. To calculate these images, we used the brain activation maps for sessions 2–4, in an identical manner to our previous HDBR and HDBR+CO_2_ analyses (Yuan et al., 2016, 2018; Hupfeld et al., 2020a; Salazar et al., 2020, 2021). We calculated regression intercepts and slopes by using weights for each image. We determined these weights using the number of days between acquisitions for each subject. We then divided the slope images by the intercept images to create normalized slope images (i.e., slope of change with the intervention, corrected for baseline activation differences). This permitted direct comparison between rates of change in brain activation for HDBR + CO_2_ and HDBR subjects, while controlling for any baseline differences. To characterize cohort differences in these intercept, slope, and normalized slope images, we used SnPM non-parametric two sample *t*-tests with 15,000 permutations and variance smoothing = 8 mm for the whole brain analyses and variance smoothing = 2 mm for the cerebellar analyses. In addition to two HDBR subjects who were excluded due to head motion during scan acquisition, we also excluded three additional subjects from these between-group analyses because their contrast values were greater than three standard deviations above the rest of the cohort and exerted a disproportionate influence on the results. Therefore, for these between-group comparisons, *n* = 13 for the HDBR cohort.

Of note, the HDBR + CO_2_ and HDBR fMRI data were collected on different MRI scanners using slightly different acquisition parameters. The images of the HDBR subjects showed more orbitofrontal dropout than images of the HDBR + CO_2_ subjects, likely a result of the differing scanners and parameters. Additionally, the HDBR subjects participated in an exercise intervention, which was controlled for in statistical analyses. Otherwise, the behavioral and neuroimaging protocols were nearly identical for the two studies. As previously mentioned, we have already reported the neural and performance effects of HDBR on dual tasking (Yuan et al., 2016). Therefore, in the present work, we report HDBR results only for exploratory examination of group differences between HDBR + CO_2_ and HDBR.

#### SANS vs. Non-SANS Comparisons Within the HDBR + CO_2_ Cohort

We conducted exploratory analyses of the HDBR+CO_2_ cohort between subjects who presented signs of SANS (“SANS,” *n* = 5) and those who did not (“non-SANS,” *n* = 6). We used SPM parametric two sample *t*-tests for these analyses. Non-parametric testing was not possible because fewer than 500 permutation combinations exist for these sample sizes. We tested for between-group differences in intercept activation, slope of activation change, and normalized slope of activation change during performance of the TAP, COUNT, and DUAL tasks, as well as the intercept, slope, and normalized slope of DTCost.

## Results

### Arterialized P_a_CO_2_ During HDBR + CO_2_

Subjects had no significant increase in P_a_CO_2_ from pre- to post- HDBR + CO_2_ (*p* > 0.05), with a mean increase of 1.2 mmHg (–0.2 to 2.5 mmHg 95% CI). Further arterial blood gas analyses have been previously reported (Laurie et al., 2020).

### Behavioral Performance

We detected no significant effects of time during the intervention (HDBR or HDBR + CO_2_) or during recovery for any of the dual task performance measures. Performance did not change with repeated exposure to the task or throughout the duration of either intervention. There was an effect of group (*p* = 0.015) during bed rest for TAP accuracy; the HDBR subjects had higher accuracy than HDBR + CO_2_ subjects, although TAP accuracy did not change significantly throughout the course of the intervention for either cohort. No other between-group differences were observed for behavioral performance.

### Functional Magnetic Resonance Imaging

#### Neural Response to Dual Task at Baseline

**FIGURE 4:**
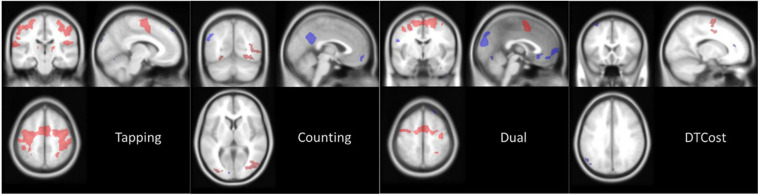
Main effect of task at baseline. Average brain activation at session 2 (BDC-7) for the HDBR + CO_2_ cohort. Contrast images are thresholded at *p* < 0.001 with a threshold of *k* = 10 for cerebral cluster and *k* = 6 for cerebellar clusters. Red areas are clusters of activation and blue areas are clusters of deactivation, both relative to values at rest.

At baseline (i.e., session 2), the main effect of each task relative to rest revealed that the brain activity of the HDBR + CO_2_ cohort was in the expected regions (Figure 4). When subjects performed the TAP task, activation occurred throughout the motor and premotor cortices, as well as much of the cerebellum. Performing the COUNT task elicited activation primarily in visual regions, including the occipital lobe. During performance of the DUAL task, areas that were involved in the TAP and COUNT single tasks were either activated or deactivated.

A positive DTCost for a region indicates that brain activation was greater during performance of the DUAL task than the combined activation during performance of the single TAP and COUNT tasks. A negative DTCost for a region indicates that activation was less during performance of the DUAL task than the combined activation during performance of the single TAP and COUNT tasks. Areas of the brain that were deactivated during the performance of the TAP or COUNT tasks at baseline (Figure 4) exhibited negative DTCost, i.e., this deactivation was greater during performance of the DUAL task than the combined deactivation of the area during the TAP and COUNT single tasks. Only areas activated during the motor TAP task saw increased activation during the DUAL task, as has been consistently reported in the literature (Herath et al., 2001; Szameitat et al., 2002; Hartley et al., 2011; Holtzer et al., 2011). This indicates that areas related to the COUNT task exhibit decreased activation during the DUAL task. Furthermore, while fewer TAP-related brain areas were activated during performance of the DUAL task, these regions were activated more than they were during performance of the TAP single task. Areas of the brain that were deactivated during performance of both single tasks were deactivated to a greater degree during the DUAL task.

#### Time Course of Change in Neural Response to HDBR + CO_2_

**FIGURE 5:**
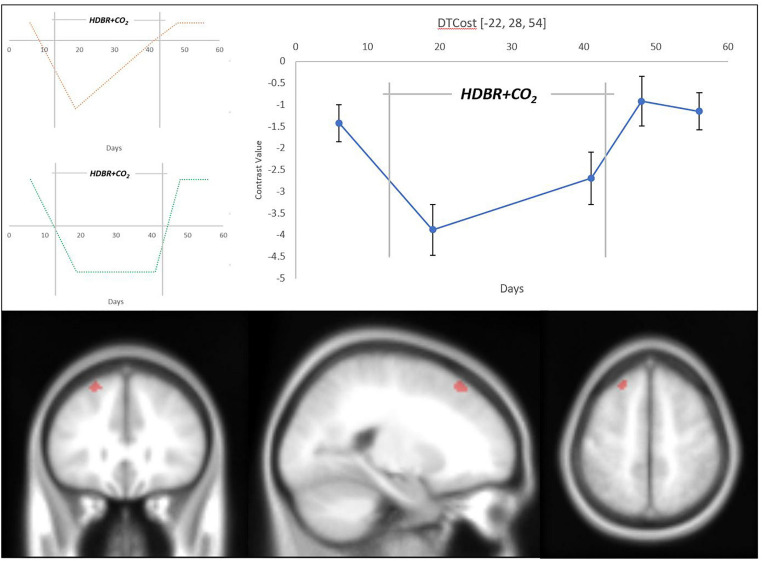
Longitudinal change in DTCost of brain activation associated with HDBR + CO_2_. (Top Left) Hypothesized models of longitudinal instant change (orange) and instant change with recovery in-HDBR (green). (Top Right) Exhibited pattern of longitudinal DTCost of brain activation change in the left superior frontal gyrus (blue). Analyses were conducted at an uncorrected alpha level of *p* < 0.0001. (Bottom) Left superior frontal gyrus cluster that showed significant longitudinal change associated with HDBR + CO_2_.

We detected changes in multiple brain regions that adhered to our hypothesized models of longitudinal change (Figure 5 and Table 2). Each of these clusters followed a clear pattern of recovery back to baseline DTCost levels after an initial HDBR + CO_2_-induced change. A visual assessment of contrast values for all clusters indicated that recovery may have started during HDBR + CO_2_, potentially indicating adaptation to the intervention.

**TABLE 2:** Longitudinal change results.

				**MNI coordinates**		
**Hypothetical model**	**Region label**	**Extent**	***t*-value**	***x***	***y***	***z***
Instant decrease	L superior frontal gyrus	38	5.003	−22	28	54
Decrease with Recovery in HDBR + CO_2_	L superior frontal gyrus	15	4.606	−24	28	52
	R cerebellum (Lobule VIIb)	11	5.119226	14	−76	−55
	L cerebellum (Crus 1)	6	4.364	−40	−54	−29

*Significant regions (*p* < 0.0001) of longitudinal change and recovery that followed the hypothesized models of change depicted in Figure 3.*

Changes in the superior frontal gyrus followed our hypothesized “instant decrease with recovery in HDBR + CO_2_” model of change. The DTCost in this cluster, assessed by changes in contrast values, was negative at all sessions, and decreased with duration in HDBR + CO_2_, suggesting that brain activation during performance of the single TAP and COUNT tasks remained higher than activation during performance of the DUAL task, and that these differences in activation during the single and dual tasks are exacerbated by HDBR + CO_2_ (Supplementary Figure 2).

Two cerebellar clusters also followed our “instant decrease with recovery in- HDBR + CO_2_” model. DTCost in these clusters was initially positive and became negative during HDBR + CO_2_ before recovering to positive baseline levels (Supplementary Figure 1), indicating that, at baseline, the cerebellum is less activated during performance of the single TAP and COUNT tasks than it is during performance of the DUAL task, and that this trend reverses during HDBR + CO_2_, at least upon initial exposure.

#### Correlations Between Behavioral and Neural Changes Associated With HDBR + CO_2_

There were no significant associations between pre-to-post bed rest changes in DTCost of brain activation and pre-to-post bed rest changes in behavioral measures or their DTCost.

#### Comparisons of Neural Changes Associated With HDBR and HDBR + CO_2_

**FIGURE 6:**
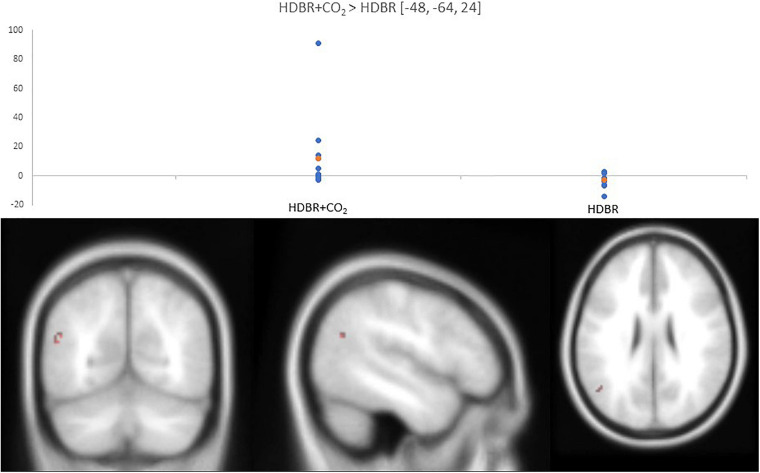
Differences in normalized slope of DTCost of brain activation between HDBR + CO_2_ and HDBR. (Top) Values for normalized slope of DTCost of brain activation in the left middle temporal gyrus for the HDBR + CO_2_ (left) and HDBR (right) cohorts. Blue, individual values. Orange, average values. Analyses were conducted at an uncorrected alpha level of *p* < 0.0001. (Bottom) Left middle temporal gyrus cluster that exhibited a significant difference in normalized slope of DTCost of brain activation between the HDBR and HDBR + CO_2_ cohorts.

The DTCost intercept values were much larger in the HDBR cohort than the HDBR + CO_2_ cohort. The normalized slope of change in one cluster in the left middle temporal gyrus was significantly different between the HDBR + CO_2_ and HDBR cohorts; the DTCost of brain activation increased within the HDBR + CO_2_ cohort and decreased in the HDBR cohort (Figure 6 and Table 3).

**TABLE 3:** HDBR vs. HDBR + CO_2_ between-group results.

				**MNI coordinates**		
**Contrast**	**Region label**	**Extent**	***t*-value**	***x***	***y***	***z***
HDBR + CO_2_ > HDBR	L middle temporal gyrus	13	0.326	−48	−64	24

*Cluster of significant difference in the normalized slope of DTCost of brain activation between the HDBR and HDBR + CO_2_ cohorts (*p* < 0.0001).*

#### Comparisons of SANS and Non-SANS Subjects Within the HDBR + CO_2_ Cohort

**FIGURE 7:**
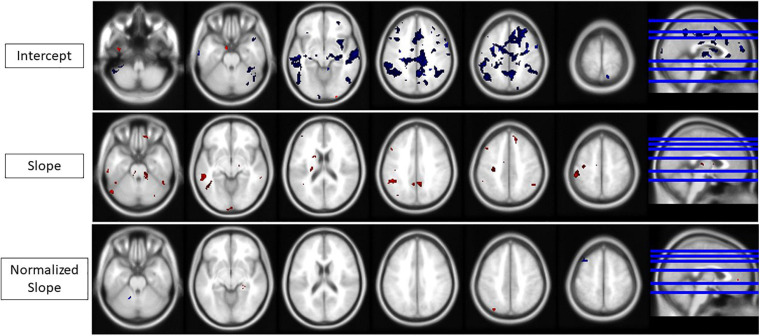
Intercept, slope, and normalized slope differences for the SANS vs. non-SANS cohorts. All areas of change for any task or DTCost. Red, SANS activation > non-SANS activation. Blue, SANS activation < non-SANS activation. (Top) Intercept differences. (Middle) Slope differences. (Bottom) Normalized slope differences. Analyses were conducted at an uncorrected alpha level of *p* < 0.001.

HDBR + CO_2_ subjects who exhibited signs of SANS had lower levels of baseline (i.e., intercept) activation during performance of all tasks (TAP, COUNT, DUAL) and lower DTCost than the HDBR + CO_2_ subjects who did not exhibit signs of SANS. The individuals with signs of SANS also had higher magnitude slopes of activation change during bed rest for all three tasks. More clusters exhibited intercept differences than slope differences for all tasks and for DTCost (Figure 7), indicating that the SANS and non-SANS subjects had different task-based activation levels before the intervention began. Before the data were normalized to intercept values, the slopes of DTCost of brain activation were similar between the two cohorts, with no significant clusters in which SANS subjects had more positive slopes than non-SANS subjects and just one in which the SANS subjects had more negative slopes. After data were normalized to intercept values, some slope differences remained; the SANS subjects had higher magnitude slopes of change than the non-SANS subjects in two temporal lobe clusters and lower slopes of change than non-SANS subjects in one frontal cluster (Figure 7 and Table 4). We recently reported a significant group (SANS, non-SANS) × time interaction for accuracy during the TAP (*p* = 0.0008) and DUAL (*p* = 0.0069) tasks, and for DUAL reaction time (*p* = 0.0011) during the intervention (Lee et al., 2019a). Additionally, we identified a group × time interaction (*p* = 0.0314) for the DTCost of reaction time during the intervention. During the recovery period after HDBR + CO_2_, we also found a significant group x time interaction for accuracy during the TAP task (*p* = 0.0098) (Supplementary Table 1).

**TABLE 4:** SANS vs. non-SANS between-group results.

					**MNI Coordinates**		
**Task**	**Contrast**	**Region label**	**Extent**	***t*-value**	***x***	***y***	***z***
TAP	SANS > non-SANS	R parahippocampal gyrus	10	7.874	24	−32	−10
		L inferior parietal lobule	24	6.642	−30	−78	48
		R superior temporal gyrus	18	6.073	56	−8	4
DUAL	SANS > non-SANS	L middle orbital gyrus	11	6.366435	−46	44	2
		L middle orbital gyrus	10	5.915862	−4	46	2
	SANS < non-SANS	L middle frontal gyrus	45	15.004	−32	18	58
DTCost	SANS > non-SANS	L hippocampus	10	9.127	−30	−14	−16
		L superior temporal gyrus	42	7.761	−62	−10	12
	SANS < non-SANS	R superior frontal gyrus	14	7.616	36	62	12

*Areas of significant difference (*p* < 0.001) in normalized slope of activation and DTCost change between the HDBR + CO_2_ subjects who did and did not present signs of SANS.*

## Discussion

### Key Findings

The HDBR subjects had greater accuracy during the TAP task than the HDBR + CO_2_ subjects did, but overall TAP performance did not change for either cohort during the interventions. Multiple brain regions exhibited longitudinal changes with HDBR + CO_2_ that followed our hypothesized models and returned toward pre-bed rest levels either during or after HDBR + CO_2_. We also found one left middle temporal gyrus cluster that exhibited increasing DTCost in HDBR + CO_2_ and decreasing DTCost in HDBR. Additionally, subjects who showed signs of SANS and subjects who did not show signs of SANS differed in a number of measures, including baseline activation levels and slope of activation change during HDBR + CO_2_. Between these cohorts, we also identified several group × time effects on DTCost of reaction time during HDBR × CO_2_ and TAP accuracy during the recovery period. The small sample size and uncorrected results of this pilot study limit its interpretability. The small sample size may have precluded us from detecting meaningful, but not statistically significant, brain-behavior associations and larger scale future studies should be conducted to confirm the present findings.

### Arterialized P_a_CO_2_ Changes During HDBR + CO_2_

We observed no significant increases in P_a_CO_2_ from pre- to post-bed rest. Spirometry measurements collected in HDBR + CO_2_ subjects (Laurie et al., 2019) and during spaceflight (Prisk et al., 2006) have previously shown no changes in end-tidal CO_2_ (P_ET_CO_2_), a surrogate for arterial CO_2_ measurement. It is also notable that previous reports from the HDBR + CO_2_ investigation have shown that respiratory acidosis, via elevated CO_2_ levels, never developed. There was, however, a metabolic alkalosis that persisted until 13 days after the completion of bed rest (Laurie et al., 2020).

### Changes in Dual Tasking Behavior During HDBR and HDBR + CO_2_

We identified several dual task performance changes that were associated with either HDBR (Yuan et al., 2016) or HDBR + CO_2_. The HDBR + CO_2_ cohort’s accuracy during the TAP task was lower than that of the HDBR cohort, but neither cohort showed any significant change during the intervention. There were no differences in post-intervention recovery patterns. Thus, as was previously noted in studies of elevated CO_2_ (Scully et al., 2019) and HDBR + CO_2_ (Basner et al., 2018), the present data do not indicate changes in cognitive performance with either elevated CO_2_ or bed rest. These recent studies also report few motor performance changes, in contrast with early case studies of cognitive-motor dual tasking during spaceflight (Manzey et al., 1995, 1998; Manzey and Lorenz, 1998b). This lack of observable change could be due to additive effects of CO_2_ with HDBR on neural compensation, the level of difficulty of the tasks themselves (i.e., the tasks used here could have been too easy for participants), or because of the challenge of adapting to an HDBR + CO_2_ environment is less demanding than adaptation to spaceflight.

### Changes in Neural Correlates of Dual Tasking During HDBR + CO_2_

#### Time Course of Change in Neural Response to HDBR + CO_2_

Baseline DTCost of brain activation was negative in the cerebrum and positive in the cerebellum; values in both regions decreased once HDBR + CO_2_ began and then returned to baseline levels after HDBR + CO_2_. We previously found that HDBR was associated with widespread longitudinal increases in DTCost of brain activation (Yuan et al., 2016) whereas, in HDBR + CO_2_, we found the opposite pattern. The cluster in the left superior frontal gyrus exhibited a negative DTCost at baseline that decreased with HDBR + CO_2_ and eventually returned to baseline, with recovery potentially beginning during HDBR + CO_2_. Cerebellar regions displayed a positive baseline DTCost that became negative during HDBR + CO_2_ and then returned to baseline values, with an even stronger recovery pattern beginning while still in HDBR + CO_2_.

Work by Manzey et al. (1995) and Manzey and Lorenz (1998b) has demonstrated that dual tasking performance measures decline after astronauts begin spaceflight and then begin to recover while still in space. We previously reported comparable recovery effects in the neural correlates of vestibular stimulation during HDBR + CO_2_ (Hupfeld et al., 2020a) and, in the present study, we observed similar patterns with dual tasking brain activity during HDBR + CO_2_. To date, there have been few fMRI studies after spaceflight. Those that have been conducted (Demertzi et al., 2016; Pechenkova et al., 2019) have been unable to follow changes in-flight due to technological limitations inherent to the modality. We recently reported that changes in structural brain metrics within the cerebellum, including fractional anisotropy, were greater after short duration spaceflight than after longer duration spaceflight (Lee et al., 2019b). This could indicate that the brain accommodates to the novel environment of spaceflight and begins to return to its normal structure and physiology during longer flights. Alternatively, some changes in brain structure, including increased ventricular volume, recover little – even when measured multiple months postflight (Van Ombergen et al., 2019; Hupfeld et al., 2020b). This potential variation in recovery for different structures underscores the importance of further study of the brain’s recovery to baseline structure and function during or after spaceflight. The inability to collect fMRI during spaceflight makes bed rest interventions critical for understanding this possible neural adaptation during spaceflight.

#### Brain-Behavior Changes Associated With HDBR + CO_2_

We previously reported positive associations between changes in DUAL reaction time in the HDBR cohort and activation changes in frontal, parietal, temporal, and occipital regions of the brain (Yuan et al., 2016), i.e., as brain activation increased in these regions, performance worsened. Increases in DTCost of reaction time were also associated with increasing DTCost of brain activation in these subjects (Yuan et al., 2016). In the current study, we found no significant brain-behavior correlations. The prior SPACECOT study also showed that the addition of CO_2_ lessened the motor performance decrement normally seen with HDBR interventions (Basner et al., 2018). Thus, it could be that CO_2_ mitigates some of the negative cognitive effects induced by other spaceflight factors.

#### Comparisons of Neural Changes Associated With HDBR and HDBR + CO_2_

The HDBR + CO_2_ cohort had more brain regions with a reduced slope of DTCost change than the HDBR cohort did. However, after normalization for intercept (i.e., baseline) values, only one cluster of DTCost slope difference in the left middle temporal gyrus remained. The middle temporal gyrus is associated with comprehension of symbolic gestures and actions (Kable et al., 2005; Kubiak and Króliczak, 2016; van Kemenade et al., 2019) and has previously been reported to show altered activity during dual task and attention-specific paradigms (Mizuno et al., 2012). The left middle temporal gyrus exhibited a positive normalized slope of DTCost in the HDBR + CO_2_ cohort and a negative normalized slope of DTCost in the HDBR cohort, i.e., DTCost of brain activation within the cluster increased with HDBR + CO_2_ and decreased with HDBR. A potential explanation for this finding is that exposure to elevated levels of CO_2_ may reduce connectivity within the default mode network (Xu et al., 2011). Alternatively, this finding could be a result of the increased cerebral perfusion associated with hypercapnia (Brian, 1998), as BOLD signal is directly proportional to the amount of deoxygenated blood flow to a region. Furthermore, recent work found that 14 months at the German Neumayer III station in Antarctica resulted in reduced hippocampal volume that correlated with spatial mental rotation performance (Stahn et al., 2019). Thus, it could also be that differences in DTCost in the left middle temporal gyrus (part of the parahippocampal cortex) are due in part to the social isolation and environmental deprivation of HDBR participation. This may indicate that this observed change in parahippocampal activation with HDBR + CO_2_ could be a compensatory response related to possible structural declines associated with the intervention. This hypothesis would also support our recent finding that greater HDBR + CO_2_-related increases in activation of multiple cortical regions, including middle temporal gyrus, are associated with better maintenance of spatial working memory (Salazar et al., 2020).

With similarly few behavioral changes and substantially more brain-behavior correlations in the HDBR cohort, the present findings, those from the SPACECOT study (Basner et al., 2018), and those from our previous work (Hupfeld et al., 2020a) suggest a compensatory response during HDBR + CO_2_ that reduces the development of behavioral changes during the intervention. Similar compensatory responses, with increased levels of generalized physiological activation (e.g., sympathetic, musculoskeletal, and heart rate responses) in place of performance decrement, have been reported in various situations (Hockey, 2010) and it is possible that more concerted and directed attention during these tasks could also lead to further increases in neural activation and preservation of task performance. Compensatory changes with structural neurological decline have been seen in older adults (Reuter-Lorenz and Lustig, 2005; Seidler et al., 2010; Zahodne and Reuter-Lorenz, 2019), a population that is sometimes used to parallel the potential effects of spaceflight (Hupfeld et al., 2021). These compensatory patterns of change, as well as functional patterns that result in behavioral change, are also seen in HDBR and HDBR + CO_2_ studies of sleep and adaptation (Hupfeld et al., 2020a). In HDBR, a similar mechanism of compensation –without complete performance preservation—could be at work, as reaction time increased with increased activation in numerous regions of the brain. However, the additional stress and nervous arousal produced by exposure to elevated CO_2_ levels could also be augmenting this response in HDBR + CO_2_ (Guyenet et al., 2010).

#### SANS vs. Non-SANS Comparisons Within the HDBR+CO_2_ Cohort

The present study is the first to report that bed rest interventions can induce signs of SANS (Laurie et al., 2019). Exploratory analyses of the HDBR+CO_2_ cohort revealed multiple differing patterns of behavioral change between the SANS and non-SANS subjects. However, we identified few differences in the normalized slope of task-based activation change and DTCost between these two cohorts, indicating differing levels of neural compensation between the two cohorts. Interestingly, SANS subjects also exhibited lower intercept values and higher slope values than the non-SANS subjects for task-based activation and DTCost throughout the brain. This raises the possibility that baseline values may affect the ability of a brain region to respond and adapt to a stressor, and it has been suggested that genetic differences and/or B-vitamin status might play a role in susceptibility to SANS (Smith and Zwart, 2018). We have previously reported (Lee et al., 2019a) that the HDBR + CO_2_ subjects who developed signs of SANS were more visually dependent in their perception of stimuli than subjects who did not develop signs of SANS, indicated by their higher “frame effect” and lower response variability during a rod and frame test. This visual dependence is a potential reason for the differential response to dual tasking in HDBR + CO_2_ between SANS and non-SANS subjects. These behavioral and activation differences between the five SANS subjects and the six non-SANS subjects could also be contributing to the overall lack of normalized slope differences between HDBR + CO_2_ and HDBR cohorts.

The signs of SANS that we observed in this study indicate that HDBR+CO_2_ is a useful spaceflight analog, but further investigation is certainly necessary. Larger sample sizes would allow for better characterization of individual differences in factors that contribute to the development of SANS and for a better understanding of how HDBR + CO_2_ interacts with SANS risk.

### Limitations

Limitations of this pilot study include a small sample size, lack of a control group, and differences in fMRI acquisition parameters and timing intervals between the HDBR and HDBR + CO_2_ cohorts. This project was conducted as a part of a large multi-investigator campaign, which greatly limited the time allotted for our task. This, in turn, limited the amount of data that could be acquired and analyzed. While the use of linear mixed models in our slope comparisons enabled us to compare both interventions despite their differing timelines, it also assumed linear change over time. Additionally, our exclusion of the first session to control for practice effects does not fully account for the learning that occurred over time, but was consistent with the methodology that has been previously used in our prior analyses (Yuan et al., 2016). The 13 HDBR subjects also underwent a concurrent exercise intervention, which was included as a covariate of no interest in all statistical analyses that involved the HDBR cohort; exercise has been shown to modulate brain changes during recovery following HDBR (Koppelmans et al., 2018). The use of exercise as a covariate of no interest has likely limited our statistical significance, as all included HDBR subjects participated in exercise while none of the HDBR + CO_2_ subjects did. small sample size necessitated the use of uncorrected *p*-values to assess statistical significance. In light of these limitations, this pilot campaign should act as a framework for larger studies in the future. Even with the addition of elevated CO_2_ levels, HDBR + CO_2_ does not mimic all of the stimuli present on the ISS, including space radiation and sleep disruption (Cooper, 1996), and it is difficult to establish the degree of environmental replication that exists with these analogs. The effects of microgravity on brain activation are also task-dependent, so these results may not be generalizable to all motor or cognitive-motor tasks (Cheron et al., 2006, 2014).

Despite these limitations, the present work demonstrates that differences occur with the addition of CO_2_ to a standard HDBR analog. Future studies should aim to validate our preliminary findings and to further characterize the time course of bed rest-related brain and behavioral changes, the quality of HDBR as a spaceflight analog, the additive effects of elevated CO_2_, the potential for neural compensation, and the individual differences in factors that contribute to SANS risk and symptoms.

## Conclusion

We report preliminary findings of performance and brain activity responses during cognitive-motor dual tasks associated with 30 days of HDBR + CO_2_. Minimal performance changes were associated with HDBR + CO_2_, similar to our previous findings with HDBR under normal atmospheric CO_2_ conditions. Some brain regions followed a pattern of decreasing activation with HDBR + CO_2_ and returned to baseline levels, with recovery appearing to start during bed rest. Minimal differences were appreciated in the normalized slopes of the DTCost brain activation between the HDBR + CO_2_ and HDBR subjects. This lack of difference could be due to differences in HDBR + CO_2_ subjects who did and did not develop signs of SANS, as we identified several differences in dual task brain activity for the SANS versus non-SANS subjects. Thus, the present work demonstrates that elevated CO_2_ in combination with HDBR likely affects brain function and behavioral performance differently than HDBR under atmospheric CO_2_ conditions. We are currently collecting similar MRI and behavioral metrics from astronauts before and after long-duration ISS missions (Koppelmans et al., 2013), in part to directly compare dual task brain activity and performance change between astronauts and bed rest subjects.

## Data Availability Statement

The datasets presented in this article are not readily available because they are held, pending request, in NASA’s data archives. Requests to access the datasets should be directed to APM, ajitkumar.p.mulavara@nasa.gov.

## Ethics Statement

The studies involving human participants were reviewed and approved by the Ethical Commission of the Arztekammer Nordrhein (HDBR + CO_2_) and the Institutional Review Boards of the University of Florida (HDBR + CO_2_), University of Michigan (HDBR), University of Texas-Medical Branch (HDBR), and NASA Johnson Space Center (HDBR and HDBR + CO_2_). The patients/participants provided their written informed consent to participate in this study.

## Author Contributions

ADM analyzed the fMRI and behavioral data, created the figures and tables, and was the primary author of the manuscript. KH assisted with data analyses, editing of figures and tables, and the manuscript writing and editing. JL and EM collected and managed data and participated in manuscript preparation. NB and YD collected and analyzed the data. IK participated in project design and software development. JB, APM, and RS designed the project, secured the funding, and led the interpretation and discussion of the results. All the authors participated in revision of the manuscript.

## Conflict of Interest

The authors declare that the research was conducted in the absence of any commercial or financial relationships that could be construed as a potential conflict of interest.

## Publisher’s Note

All claims expressed in this article are solely those of the authors and do not necessarily represent those of their affiliated organizations, or those of the publisher, the editors and the reviewers. Any product that may be evaluated in this article, or claim that may be made by its manufacturer, is not guaranteed or endorsed by the publisher.

## Supplementary Material

Supplementary Figure 1 Longitudinal change in DTCost of brain activation (Cerebellum). Cerebellar clusters that exhibited longitudinal patterns of change matching the hypothesized Instant Decrease with Recovery in-Bed Rest Model. Analyses were conducted at an uncorrected alpha level of *p* < 0.0001.

Supplementary Figure 2 Longitudinal change in brain activation (left superior frontal gyrus). Comparison of longitudinal changes in DTCost and its constituent TAP, COUNT, and DUAL tasks within the left superior frontal gyrus.

## References

[B1] Adcock R. A. Constable R. T. Gore J. C. Goldman-Rakic P. S. (2000). Functional neuroanatomy of executive processes involved in dual-task performance. *Proc. Natl. Acad. Sci. U.S.A.* 97 3567–3572. 10.1073/pnas.060588897 10725387 PMC16280

[B2] Alperin N. Bagci A. M. (2018). Spaceflight-induced visual impairment and globe deformations in astronauts are linked to orbital cerebrospinal fluid volume increase. *Acta Neurochir. Suppl.* 126 215–219. 10.1007/978-3-319-65798-1_44 29492564

[B3] Ashburner J. Barnes G. Chen C.-C. Daunizeau J. Flandin G. Friston K. (2016). *SPM12 Manual.* Available online at: http://web.mit.edu/spm_v12/manual.pdf (accessed January 1, 2018).

[B4] At’kov O. I. Bednenko V. S. (1992). *Hypokinesia and Weightlessness: Clinical and Physiologic Aspects.* Madison, WI: International Universities Press.

[B5] Basner M. Nasrini J. Hermosillo E. McGuire S. Dinges D. F. Moore T. M. (2018). Effects of -12∘ head-down tilt with and without elevated levels of CO2 on cognitive performance: the SPACECOT study. *J. Appl. Physiol.* 124 750–760. 10.1152/japplphysiol.00855.2017 29357516

[B6] Basner M. Stahn A. C. Nasrini J. Dinges D. F. Moore T. M. Gur R. C. (2021). Effects of head-down tilt bed rest plus elevated CO2 on cognitive performance. *J. Appl. Physiol.* 130 1235–1246. 10.1152/japplphysiol.00865.2020 33630672 PMC8262780

[B7] Battisti-Charbonney A. Fisher J. Duffin J. (2011). The cerebrovascular response to carbon dioxide in humans. *J. Physiol.* 589 3039–3048. 10.1113/jphysiol.2011.206052 21521758 PMC3139085

[B8] Bock O. Weigelt C. Bloomberg J. J. (2010). Cognitive demand of human sensorimotor performance during an extended space mission: a dual-task study. *Aviat. Space Environ. Med.* 81 819–824. 10.3357/asem.2608.2010 20824987

[B9] Brian J. E. (1998). Carbon dioxide and the cerebral circulation. *Anesthesiology* 88 1365–1386. 10.1097/00000542-199805000-00029 9605698

[B10] Brunstetter T. (2017). *Introduction to Spaceflight Associated Neuro-ocular Syndrome (SANS) and its Risk to NASA Astronauts.* Available online at: https://ntrs.nasa.gov/archive/nasa/casi.ntrs.nasa.gov/20170009173.pdf (accessed January 4, 2018).

[B11] Caprihan A. Sanders J. A. Cheng H. A. Loeppky J. A. (1999). Effect of head-down tilt on brain water distribution. *Eur. J. Appl. Physiol.* 79 367–373. 10.1007/s004210050522 10090638

[B12] Castro D. Keenaghan M. (2019). “Arterial blood gas,” in *StatPearls*, (Treasure Island, FL: StatPearls Publishing). Available online at: http://www.ncbi.nlm.nih.gov/books/NBK536919/ (accessed June 28, 2019). 30725604

[B13] Cheron G. Leroy A. De Saedeleer C. Bengoetxea A. Lipshits M. Cebolla A. (2006). Effect of gravity on human spontaneous 10-Hz electroencephalographic oscillations during the arrest reaction. *Brain Res.* 1121 104–116. 10.1016/j.brainres.2006.08.098 17034767

[B14] Cheron G. Leroy A. Palmero-Soler E. De Saedeleer C. Bengoetxea A. Cebolla A.-M. (2014). Gravity influences top-down signals in visual processing. *PLoS One* 9:e82371. 10.1371/journal.pone.0082371 24400069 PMC3882212

[B15] Chumbley J. Worsley K. Flandin G. Friston K. (2010). Topological FDR for neuroimaging. *Neuroimage* 49 3057–3064. 10.1016/j.neuroimage.2009.10.090 19944173 PMC3221040

[B16] Cooper H. S. (1996). The loneliness of the long-duration astronaut. *Air Space* 11 37–45. 11540537

[B17] D’Esposito M. Detre J. A. Alsop D. C. Shin R. K. Atlas S. Grossman M. (1995). The neural basis of the central executive system of working memory. *Nature* 378 279–281. 10.1038/378279a0 7477346

[B18] Demertzi A. Van Ombergen A. Tomilovskaya E. Jeurissen B. Pechenkova E. Di Perri C. (2016). Cortical reorganization in an astronaut’s brain after long-duration spaceflight. *Brain Struct. Funct.* 221 2873–2876. 10.1007/s00429-015-1054-3 25963710 PMC4884200

[B19] Diedrichsen J. (2006). A spatially unbiased atlas template of the human cerebellum. *Neuroimage* 33 127–138. 10.1016/j.neuroimage.2006.05.056 16904911

[B20] Diedrichsen J. Balsters J. H. Flavell J. Cussans E. Ramnani N. (2009). A probabilistic MR atlas of the human cerebellum. *Neuroimage* 46 39–46. 10.1016/j.neuroimage.2009.01.045 19457380

[B21] Dux P. E. Tombu M. N. Harrison S. Rogers B. P. Tong F. Marois R. (2009). Training improves multitasking performance by increasing the speed of information processing in human prefrontal cortex. *Neuron* 63 127–138. 10.1016/j.neuron.2009.06.005 19607798 PMC2713348

[B22] Erickson K. I. Colcombe S. J. Wadhwa R. Bherer L. Peterson M. S. Scalf P. E. (2007). Training-induced functional activation changes in dual-task processing: an FMRI study. *Cereb. Cortex* 17 192–204. 10.1093/cercor/bhj137 16467562

[B23] Friston K. J. Ashburner J. Frith C. D. Poline J.-B. Heather J. D. Frackowiak R. S. (1995). Spatial registration and normalization of images. *Hum. Brain Mapp.* 3 165–189. 10.1002/hbm.460030303

[B24] Guyenet P. G. Stornetta R. L. Bayliss D. A. (2010). Central respiratory chemoreception. *J. Comp. Neurol.* 518 3883–3906. 10.1002/cne.22435 20737591 PMC2929977

[B25] Hartley A. A. Jonides J. Sylvester C.-Y. C. (2011). Dual-task processing in younger and older adults: similarities and differences revealed by fMRI. *Brain Cogn.* 75 281–291. 10.1016/j.bandc.2011.01.004 21320741

[B26] Herath P. Klingberg T. Young J. Amunts K. Roland P. (2001). Neural correlates of dual task interference can be dissociated from those of divided attention: an fMRI study. *Cereb. Cortex* 11 796–805. 10.1093/cercor/11.9.796 11532885

[B27] Hockey G. (2010). “Environmental stress, effects on human performance,” in *Stress Consequences: Mental, Neuropsychological and Socioeconomic*, ed. Fink G. (Cambridge, MA: Academic Press), 663–668.

[B28] Hockey G. R. J. (1986). “Changes in operator efficiency as a function of environmental stress, fatigue, and circadian rhythms,” in *Handbook of Perception and Human Performance Volume II: Cognitive Processes and Performance*, eds Boff K. R. Kaufman L. Thomas J. P. (New York, NY: John Wiley & Sons).

[B29] Holtzer R. Mahoney J. R. Izzetoglu M. Izzetoglu K. Onaral B. Verghese J. (2011). fNIRS study of walking and walking while talking in young and old individuals. *J. Gerontol. A Biol. Sci. Med. Sci.* 66 879–887. 10.1093/gerona/glr068 21593013 PMC3148759

[B30] Hupfeld K. E. McGregor H. R. Reuter-Lorenz P. A. Seidler R. D. (2021). Microgravity effects on the human brain and behavior: dysfunction and adaptive plasticity. *Neurosci Biobehav Reviews.* 122 176–189. 10.1016/j.neubiorev.2020.11.017 33454290 PMC9650717

[B31] Hupfeld K. E. Lee J. K. Gadd N. E. Kofman I. S. De Dios Y. E. Bloomberg J. J. (2020a). Neural correlates of vestibular processing during a spaceflight analog with elevated carbon dioxide (CO_2_): a pilot study. *Front. Syst. Neurosci.* 13:80. 10.3389/fnsys.2019.00080 31998084 PMC6965349

[B32] Hupfeld K. E. McGregor H. R. Lee J. K. Beltran N. E. Kofman I. S. De Dios Y. E. (2020b). The impact of 6 and 12 months in space on human brain structure and intracranial fluid shifts. *Cereb. Cortex Commun.* 1:tgaa023. 10.1093/texcom/tgaa023 32864615 PMC7446230

[B33] Kable J. W. Kan I. P. Wilson A. Thompson-Schill S. L. Chatterjee A. (2005). Conceptual representations of action in the lateral temporal cortex. *J. Cogn. Neurosci.* 17 1855–1870. 10.1162/089892905775008625 16356324

[B34] Koppelmans V. Bloomberg J. J. Dios Y. E. D. Wood S. J. Reuter-Lorenz P. A. Kofman I. S. (2017). Brain plasticity and sensorimotor deterioration as a function of 70 days head down tilt bed rest. *PLoS One* 12:e0182236. 10.1371/journal.pone.0182236 28767698 PMC5540603

[B35] Koppelmans V. Bloomberg J. J. Mulavara A. P. Seidler R. D. (2016). Brain structural plasticity with spaceflight. *NPJ Microgravity* 2:2. 10.1038/s41526-016-0001-9 28649622 PMC5460234

[B36] Koppelmans V. Erdeniz B. De Dios Y. E. Wood S. J. Reuter-Lorenz P. A. Kofman I. (2013). Study protocol to examine the effects of spaceflight and a spaceflight analog on neurocognitive performance: extent, longevity, and neural bases. *BMC Neurol.* 13:205. 10.1186/1471-2377-13-205 24350728 PMC3878338

[B37] Koppelmans V. Scott J. M. Downs M. E. Cassady K. E. Yuan P. Pasternak O. (2018). Exercise effects on bed rest-induced brain changes. *PLoS One* 13:e0205515. 10.1371/journal.pone.0205515 30308004 PMC6181401

[B38] Kubiak A. Króliczak G. (2016). Left extrastriate body area is sensitive to the meaning of symbolic gesture: evidence from fMRI repetition suppression. *Sci. Rep.* 6:31064. 10.1038/srep31064 27528007 PMC4985812

[B39] Lathan C. E. Newman D. J. (1997). Quantification of human performance in extreme environments. *Adv. Hum. Factors Ergon.* 21B 1005–1008.

[B40] Laurie S. S. Christian K. Kysar J. Lee S. M. C. Lovering A. T. Macias B. R. (2020). Unchanged cerebrovascular CO2 reactivity and hypercapnic ventilatory response during strict head-down tilt bed rest in a mild hypercapnic environment. *J. Physiol.* 598 2491–2505. 10.1113/JP279383 32196672

[B41] Laurie S. S. Macias B. R. Dunn J. T. Young M. Stern C. Lee S. M. (2019). Optic disc edema after 30 days of strict head-down tilt bed rest. *Ophthalmology* 126 467–468. 10.1016/j.ophtha.2018.09.042 30308219

[B42] Law J. Van Baalen M. Foy M. Mason S. S. Mendez C. Wear M. L. (2014). Relationship between carbon dioxide levels and reported headaches on the international space station. *J. Occup. Environ. Med.* 56 477–483. 10.1097/JOM.0000000000000158 24806559

[B43] Lee J. K. De Dios Y. E. Kofman I. S. Mulavara A. P. Bloomberg J. J. Seidler R. D. (2019a). Head down tilt bed rest plus elevated CO2 as a spaceflight analog: effects on cognitive and sensorimotor performance. *Front. Hum. Neurosci.* 13:355. 10.3389/fnhum.2019.00355 31680909 PMC6811492

[B44] Lee J. K. Koppelmans V. Riascos R. F. Hasan K. M. Pasternak O. Mulavara A. P. (2019b). Spaceflight-associated brain white matter microstructural changes and intracranial fluid redistribution. *JAMA Neurol.* 76 412–419. 10.1001/jamaneurol.2018.4882 30673793 PMC6459132

[B45] Manzey D. (2000). Monitoring of mental performance during spaceflight. *Aviat. Space Environ. Med.* 71 A69–A75. 10993313

[B46] Manzey D. Lorenz B. (1998a). Joint NASA-ESA-DARA study. Part three: effects of chronically elevated CO2 on mental performance during 26 days of confinement. *Aviat. Space Environ. Med.* 69 506–514. 9591623

[B47] Manzey D. Lorenz B. (1998b). Mental performance during short-term and long-term spaceflight. *Brain Res. Rev.* 28 215–221. 10.1016/s0165-0173(98)00041-1 9795225

[B48] Manzey D. Lorenz B. Poljakov V. (1998). Mental performance in extreme environments: results from a performance monitoring study during a 438-day spaceflight. *Ergonomics* 41 537–559. 10.1080/001401398186991 9557591

[B49] Manzey D. Lorenz B. Schiewe A. Finell G. Thiele G. (1995). Dual-task performance in space: results from a single-case study during a short-term space mission. *Hum. Factors* 37 667–681. 10.1518/001872095778995599 8851772

[B50] Marshall-Goebel K. Mulder E. Donoviel D. Strangman G. Suarez J. I. Venkatasubba Rao C. (2017). An international collaboration studying the physiological and anatomical cerebral effects of carbon dioxide during head-down tilt bed rest: the SPACECOT study. *J. Appl. Physiol.* 122 1398–1405. 10.1152/japplphysiol.00885.2016 28235859

[B51] Mirelman A. Maidan I. Bernad-Elazari H. Nieuwhof F. Reelick M. Giladi N. (2014). Increased frontal brain activation during walking while dual tasking: an fNIRS study in healthy young adults. *J. Neuroeng. Rehabil.* 11:85. 10.1186/1743-0003-11-85 24886198 PMC4055254

[B52] Mizuno K. Tanaka M. Tanabe H. C. Sadato N. Watanabe Y. (2012). The neural substrates associated with attentional resources and difficulty of concurrent processing of the two verbal tasks. *Neuropsychologia* 50 1998–2009. 10.1016/j.neuropsychologia.2012.04.025 22571931

[B53] Nichols T. E. Holmes A. P. (2002). Nonparametric permutation tests for functional neuroimaging: a primer with examples. *Hum. Brain Mapp.* 15 1–25. 10.1002/hbm.1058 11747097 PMC6871862

[B54] Ostwald D. Schneider S. Bruckner R. Horvath L. (2018). Random field theory-based P-values: a review of the SPM implementation. *arXiv* [Preprint]. arXiv:180804075

[B55] Pechenkova E. Nosikova I. Rumshiskaya A. Litvinova L. Rukavishnikov I. Mershina E. (2019). Alterations of functional brain connectivity after long-duration spaceflight as revealed by fMRI. *Front. Physiol.* 10:761. 10.3389/fphys.2019.00761 31333476 PMC6621543

[B56] Penny W. D. Friston K. J. Ashburner J. T. Kiebel S. J. Nichols T. E. (2011). *Statistical Parametric Mapping: The Analysis of Functional Brain Images.* New York, NY: Elsevier.

[B57] Prisk G. K. Fine J. M. Cooper T. K. West J. B. (2006). Vital capacity, respiratory muscle strength, and pulmonary gas exchange during long-duration exposure to microgravity. *J. Appl. Physiol.* 101 439–447. 10.1152/japplphysiol.01419.2005 16601306

[B58] R Core Team (2013). *R: A Language and Environment for Statistical Computing.* Vienna: R Foundation for Statistical Computing.

[B59] Reuter-Lorenz P. A. Lustig C. (2005). Brain aging: reorganizing discoveries about the aging mind. *Curr Opin Neurobiol* 15 245–251. 10.1016/j.conb.2005.03.016 15831410

[B60] Riby L. Perfect T. Stollery B. (2004). The effects of age and task domain on dual task performance: a meta-analysis. *Eur. J. Cogn. Psychol.* 16 863–891. 10.1080/09541440340000402

[B61] Romero J. E. Coupé P. Giraud R. Ta V.-T. Fonov V. Park M. T. M. (2017). CERES: a new cerebellum lobule segmentation method. *Neuroimage* 147 916–924. 10.1016/j.neuroimage.2016.11.003 27833012

[B62] Salazar A. P. Hupfeld K. E. Lee J. K. Beltran N. E. Kofman I. S. De Dios Y. E. (2020). Neural working memory changes during a spaceflight analog with elevated carbon dioxide: a pilot study. *Front. Syst. Neurosci.* 14:48. 10.3389/fnsys.2020.00048 32848641 PMC7399639

[B63] Salazar A. P. Hupfeld K. E. Lee J. K. Banker L. A. Tays G. D. Beltran N. E. (2021). Visuomotor adaptation brain changes during a spaceflight analog with elevated carbon dioxide (CO2): A Pilot Study. *Front Neural Circuits* 15:659557. 10.3389/fncir.2021.659557 and doi: 10.1037/0000143-008 34163332 PMC8215599

[B64] Satish U. Mendell M. J. Shekhar K. Hotchi T. Sullivan D. Streufert S. (2012). Is CO2 an indoor pollutant? Direct effects of low-to-moderate CO2 concentrations on human decision-making performance. *Environ. Health Perspect.* 120 1671–1677. 10.1289/ehp.1104789 23008272 PMC3548274

[B65] Schaefer S. Schumacher V. (2011). The Interplay between cognitive and motor functioning in healthy older adults: findings from dual-task studies and suggestions for intervention. *Gerontology* 57 239–246. 10.1159/000322197 20980735

[B66] Scully R. R. Basner M. Nasrini J. Lam C.-W. Hermosillo E. Gur R. C. (2019). Effects of acute exposures to carbon dioxide on decision making and cognition in astronaut-like subjects. *NPJ Microgravity* 5:17. 10.1038/s41526-019-0071-6 31240239 PMC6584569

[B67] Seidler R. D. Bernard J. A. Burutolu T. B. Fling B. W. Gordon M. T. Gwin J. T. (2010). Motor control and aging: links to age-related brain structural, functional, and biochemical effects. *Neurosci Biobehav Rev.* 34 721–733. 10.1016/j.neubiorev.2009.10.005 19850077 PMC2838968

[B68] Smith S. M. Zwart S. R. (2018). Spaceflight-related ocular changes: the potential role of genetics, and the potential of B vitamins as a countermeasure. *Curr. Opin. Clin. Nutr. Metab. Care* 21 481–488. 10.1097/MCO.0000000000000510 30169456

[B69] Stahn A. C. Gunga H.-C. Kohlberg E. Gallinat J. Dinges D. F. Kühn S. (2019). Brain changes in response to long antarctic expeditions. *N. Engl. J. Med.* 381 2273–2275. 10.1056/nejmc1904905 31800997

[B70] Strangman G. E. Sipes W. Beven G. (2014). Human cognitive performance in spaceflight and analogue environments. *Aviat. Space Environ. Med.* 85 1033–1048. 10.3357/ASEM.3961.2014 25245904

[B71] Szameitat A. J. Schubert T. Müller K. von Cramon D. Y. (2002). Localization of executive functions in dual-task performance with fMRI. *J. Cogn. Neurosci.* 14 1184–1199. 10.1162/089892902760807195 12495525

[B72] Tays G. Hupfeld K. E. McGregor H. R. Salazar A. DeDios Y. Beltran N. (2021). The effects of long duration spaceflight on sensorimotor control and cognition. *bioRxiv* [Preprint]. 10.1101/2021.06.22.449414 PMC8577506 34764856

[B73] Tombu M. Jolicoeur P. (2003). A central capacity sharing model of dual-task performance. *J. Exp. Psychol. Hum. Percept. Perform.* 29 3–18. 10.1037//0096-1523.29.1.3 12669744

[B74] van Kemenade B. M. Arikan B. E. Podranski K. Steinsträter O. Kircher T. Straube B. (2019). Distinct roles for the cerebellum, angular gyrus, and middle temporal gyrus in action-feedback monitoring. *Cereb. Cortex* 29 1520–1531. 10.1093/cercor/bhy048 29912297

[B75] Van Ombergen A. Jillings S. Jeurissen B. Tomilovskaya E. Rumshiskaya A. Litvinova L. (2019). Brain ventricular volume changes induced by long-duration spaceflight. *Proc. Natl. Acad. Sci. U.S.A.* 116 10531–10536. 10.1073/pnas.1820354116 31061119 PMC6535034

[B76] Verhaeghen P. Steitz D. W. Sliwinski M. J. Cerella J. (2003). Aging and dual-task performance: a meta-analysis. *Psychol. Aging* 18 443–460. 10.1037/0882-7974.18.3.443 14518807

[B77] Xu F. Uh J. Brier M. R. Hart J. Yezhuvath U. S. Gu H. (2011). The influence of carbon dioxide on brain activity and metabolism in conscious humans. *J. Cereb. Blood Flow Metab.* 31 58–67. 10.1038/jcbfm.2010.153 20842164 PMC3049465

[B78] Yuan P. Koppelmans V. Reuter-Lorenz P. A. De Dios Y. E. Gadd N. E. Wood S. J. (2016). Increased brain activation for dual tasking with 70-days head-down bed rest. *Front. Syst. Neurosci.* 10:71. 10.3389/fnsys.2016.00071 27601982 PMC4993791

[B79] Yuan P. Koppelmans V. Reuter-Lorenz P. De Dios Y. Gadd N. Wood S. (2018). Vestibular brain changes within 70 days of head down bed rest. *Hum. Brain Mapp.* 39 2753–2763. 10.1002/hbm.24037 29528169 PMC6033666

[B80] Zahodne L. B. Reuter-Lorenz P. A. (2019). “Compensation and brain aging: A review and analysis of evidence,” in *The Aging Brain: Functional Adaptation Across Adulthood* (Washington, DC, US: American Psychological Association), 185–216.

