# Supplementary figures and images for: Head-Down-Tilt Bed Rest With Elevated CO2: Effects of a Pilot Spaceflight Analog on Neural Function and Performance During a Cognitive-Motor Dual Task
Source: Front Physiol. 2021 Aug 25;12:654906. doi: 10.3389/fphys.2021.654906 (PMC8424013; doi:10.3389/fphys.2021.654906)

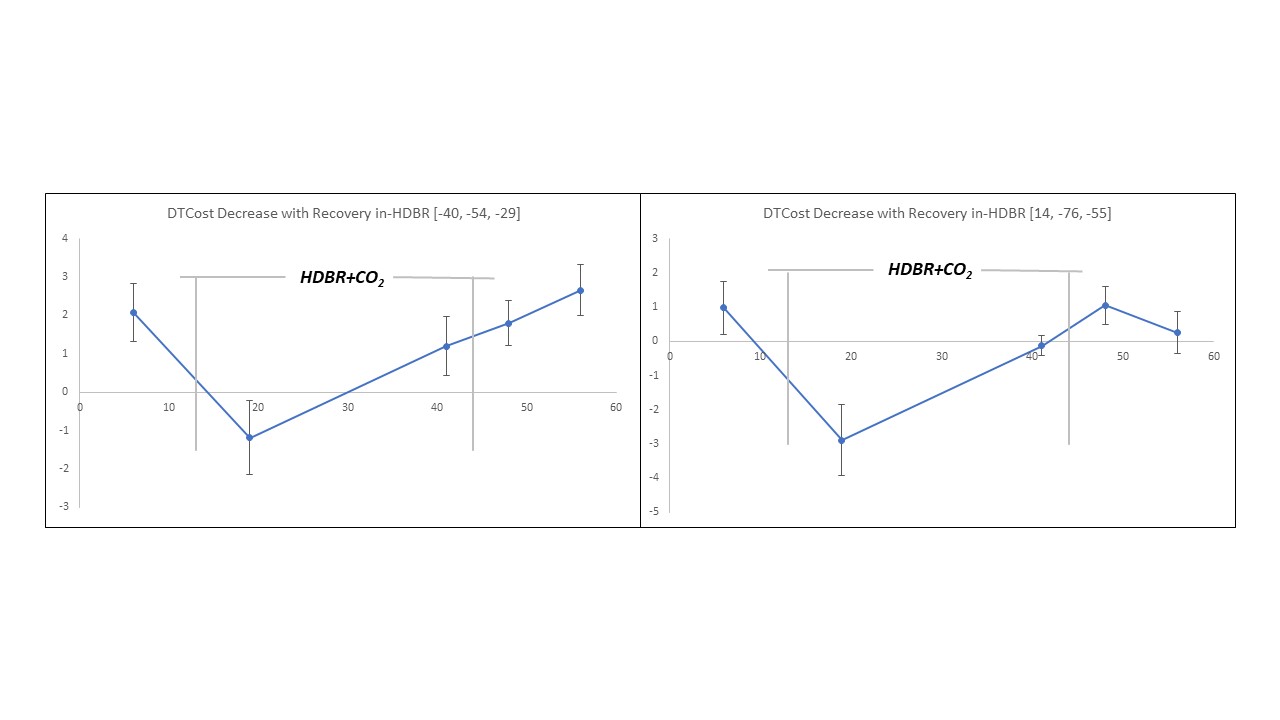

Supplement: Supplementary Figure 1 — Longitudinal change in DTCost of brain activation (Cerebellum). Cerebellar clusters that exhibited longitudinal patterns of change matching the hypothesized Instant Decrease with Recovery in-Bed Rest Model. Analyses were conducted at an uncorrected alpha level of p < 0.0001. [file Image_1.JPEG]

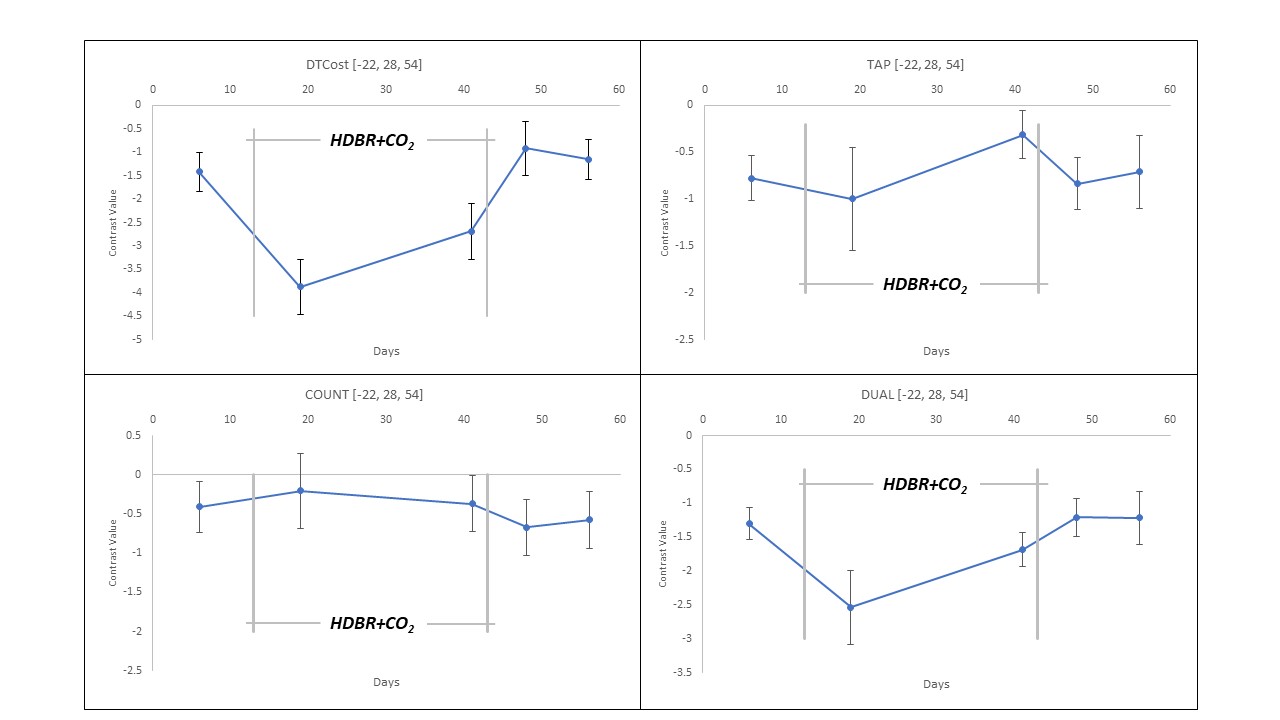

Supplement: Supplementary Figure 2 — Longitudinal change in brain activation (left superior frontal gyrus). Comparison of longitudinal changes in DTCost and its constituent TAP, COUNT, and DUAL tasks within the left superior frontal gyrus. [file Image_2.JPEG]
