# Supplementary material for: Head-Down-Tilt Bed Rest With Elevated CO2: Effects of a Pilot Spaceflight Analog on Neural Function and Performance During a Cognitive-Motor Dual Task
Source: Front Physiol. 2021 Aug 25;12:654906. doi: 10.3389/fphys.2021.654906 (PMC8424013; doi:10.3389/fphys.2021.654906)
Supplement: Supplementary file 3 [file Table_1.DOCX]

***Supplementary Material***

1. **Supplementary Figures and Tables**
   1. **Supplementary Tables**

| **SANS vs. nonSANS**  **(time x group) - intervention** | **P-value (time)** | **Std. Error** | **P-value (group)** | **Std. Error** | **P-value**  **(group x time)** | **Std. Error** |
| --- | --- | --- | --- | --- | --- | --- |
| Tap - Accuracy | 0.0014 | 0.060589 | 0.0829 | 2.166695 | 0.0008 | 0.036659 |
| Tap - Reaction Time | 0.2781 | 0.00096915 | 0.0451 | 0.01527178 | 0.1437 | 0.00058736 |
| Tap - Reaction Time SEM | 0.357 | 0.000062463 | 0.925 | 0.001716211 | 0.6899 | 0.000037801 |
| Dual - Accuracy | 0.0146 | 0.17446 | 0.0636 | 3.563272 | 0.0069 | 0.10563 |
| Dual - Reaction Time | 0.0041 | 0.00079045 | 0.2347 | 0.02324916 | 0.0011 | 0.00047833 |
| Dual - Reaction Time SEM | 0.4775 | 0.000064635 | 0.3381 | 0.001914553 | 0.5856 | 0.000039113 |
| *Accuracy DTCost* | 0.1252 | 0.00169111 | 0.1474 | 0.02369678 | 0.0736 | 0.0010258 |
| *Reaction Time DTCost* | 0.0383 | 0.00172543 | 0.6786 | 0.05015488 | 0.0314 | 0.00104413 |
| *Reaction Time SEM DTCost* | 0.3723 | 1.0449883 | 0.3538 | 0.3194587 | 0.9039 | 0.008734 |
|  |  |  |  |  |  |  |
| ***SANS vs. nonSANS**  **(time x group) - recovery*** | **P-value (time)** | **Std. Error** | **P-value (group)** | **Std. Error** | **P-value**  **(group x time)** | **Std. Error** |
| Tap - Accuracy | 0.0116 | 0.21 | 0.0649 | 5.873244 | 0.0098 | 0.129335 |
| Tap - Reaction Time | 0.9897 | 0.08426579 | 0.7419 | 0.04494538 | 0.6913 | 0.00102288 |
| Tap - Reaction Time SEM | 0.3904 | 0.000118862 | 0.6134 | 0.003421301 | 0.3437 | 0.000073205 |
| Dual - Accuracy | 0.0952 | 0.269167 | 0.0785 | 8.23586 | 0.0518 | 0.165775 |
| Dual - Reaction Time | 0.1228 | 0.00140342 | 0.0343 | 0.03908521 | 0.0813 | 0.00086434 |
| Dual - Reaction Time SEM | 0.9387 | 0.000122449 | 0.7071 | 0.003795978 | 0.9456 | 0.000075414 |
| Accuracy DTCost | 0.6792 | 0.00265225 | 0.5371 | 0.07412892 | 0.9057 | 0.00163347 |
| Reaction Time DTCost | 0.1667 | 0.00383382 | 0.0877 | 0.11304309 | 0.2383 | 0.00236118 |
| Reaction Time SEM DTCost | 0.7161 | 0.0394709 | 0.657 | 1.0497175 | 0.6802 | 0.0243094 |

**Supplementary Table 1.** SANS v. nonSANS Behavioral Results: Behavioral comparison of SANS vs. nonSANS subjects within the HDBR+CO_2_ sample.

*Indicates analyses that were not previously reported (Lee et al., 2019a)

Orange = p < 0.05; Yellow = p < 0.01
