# Supplementary material for: Head-Down-Tilt Bed Rest With Elevated CO2: Effects of a Pilot Spaceflight Analog on Neural Function and Performance During a Cognitive-Motor Dual Task
Source: Front Physiol. 2021 Aug 25;12:654906. doi: 10.3389/fphys.2021.654906 (PMC8424013; doi:10.3389/fphys.2021.654906)
Supplement: Supplementary file 4 [file Table_2.docx]

***Supplementary Material***

1. **Supplementary Text**
   1. **Medication Review**

By virtue of their participation in an inpatient study, many subjects required maintenance medication based on common, benign clinical presentations. We are not privy to complete clinical reports, so we cannot state specifically why these subjects received the medications that they did at the times that they did. A single subject was diagnosed with an ear infection and medicated accordingly. Current standards for the clinical diagnosis of otitis media do not require fever to be present, but rather middle ear effusions, otorrhea, and/or ear pain. Without the information provided in these clinical reports, it is difficult to know if a fever was present in this subject. They were given a weeklong course of topical Otalgan (benzocaine) ear drops for ear pain, followed by an 8-day course of amoxicillin. The task and imaging were performed on the first and last days of their amoxicillin course. A single subject received a dose of the antimuscarinic atropine five days prior to their subsequent acquisition. Another received a dose of the centrally active antimuscarinic scopolamine four days prior to their subsequent acquisition. Four subjects received the non-selective antihistamine Vomex (dimenhydrinate) for nausea. Three of these subjects did not have their task performance and/or imaging affected as their subsequent acquisition sessions were six days later (two subjects) and eight days later. A single subject received Vomex on the day of their BDC-13 acquisition. Regardless of medication status, the BDC-13 acquisitions for all eleven subjects were excluded from analysis to help control for learning/practice effects.

Eight of eleven subjects requested and received, at some point, one or more 400mg doses of ibuprofen and/or 500 mg doses of Paracetamol (acetaminophen), likely for muscle pains. Three of these subjects were also given the H2 selective antihistamine ranitidine, presumably to offset the risk for gastric ulcer development with NSAID use. One of these three subjects was also given chewable antacids. H2 selective antihistamines do not have central effects, so they do not pose the same concerns as H1 selective antihistamines (dimenhydrinate) or antimuscarinic agents. One subject received a dose of ibuprofen on the day of their BDC-13 acquisition, and another received a dose on the days of their R+5 and R+12 acquisitions. Muscle soreness from prolonged immobilization and physical therapy was, however, unlikely to affect task performance as the task required only movement of the right and left index fingers.
